# Muscle-Driven Predictive Physics Simulations of Quadrupedal Locomotion in the Horse

**DOI:** 10.1093/icb/icae095

**Published:** 2024-07-13

**Authors:** Pasha A van Bijlert, Thomas Geijtenbeek, Ineke H Smit, Anne S Schulp, Karl T Bates

**Affiliations:** Department of Earth Sciences, Faculty of Geosciences, Utrecht University, Vening Meinesz Building A, Princetonlaan 8A, 3584 CB Utrecht, the Netherlands; Vertebrate evolution, development and ecology, Naturalis Biodiversity Center, Darwinweg 2, 2333 CR Leiden, the Netherlands; Goatstream, Utrecht, the Netherlands; Department of Equine Musculoskeletal Biology, Faculty of Veterinary Sciences, Utrecht University, Yalelaan 112-114, 3584 CM Utrecht, the Netherlands; Department of Earth Sciences, Faculty of Geosciences, Utrecht University, Vening Meinesz Building A, Princetonlaan 8A, 3584 CB Utrecht, the Netherlands; Vertebrate evolution, development and ecology, Naturalis Biodiversity Center, Darwinweg 2, 2333 CR Leiden, the Netherlands; Department of Musculoskeletal & Ageing Science, Institute of Life Course & Medical Sciences, University of Liverpool, The William Henry Duncan Building, 6 West Derby Street, Liverpool L7 8TX, UK

## Abstract

Musculoskeletal simulations can provide insights into the underlying mechanisms that govern animal locomotion. In this study, we describe the development of a new musculoskeletal model of the horse, and to our knowledge present the first fully muscle-driven, predictive simulations of equine locomotion. Our goal was to simulate a model that captures only the gross musculoskeletal structure of a horse, without specialized morphological features. We mostly present simulations acquired using feedforward control, without state feedback (“top-down control”). Without using kinematics or motion capture data as an input, we have simulated a variety of gaits that are commonly used by horses (walk, pace, trot, tölt, and collected gallop). We also found a selection of gaits that are not normally seen in horses (half bound, extended gallop, ambling). Due to the clinical relevance of the trot, we performed a tracking simulation that included empirical joint angle deviations in the cost function. To further demonstrate the flexibility of our model, we also present a simulation acquired using spinal feedback control, where muscle control signals are wholly determined by gait kinematics. Despite simplifications to the musculature, simulated footfalls and ground reaction forces followed empirical patterns. In the tracking simulation, kinematics improved with respect to the fully predictive simulations, and muscle activations showed a reasonable correspondence to electromyographic signals, although we did not predict any anticipatory firing of muscles. When sequentially increasing the target speed, our simulations spontaneously predicted walk-to-run transitions at the empirically determined speed. However, predicted stride lengths were too short over nearly the entire speed range unless explicitly prescribed in the controller, and we also did not recover spontaneous transitions to asymmetric gaits such as galloping. Taken together, our model performed adequately when simulating individual gaits, but our simulation workflow was not able to capture all aspects of gait selection. We point out certain aspects of our workflow that may have caused this, including anatomical simplifications and the use of massless Hill-type actuators. Our model is an extensible, generalized horse model, with considerable scope for adding anatomical complexity. This project is intended as a starting point for continual development of the model and code that we make available in extensible open-source formats.

## Introduction

Locomotion in the horse (*Equus ferus caballus*) is a topic that has sparked scientific interest for centuries. A fascination for equine locomotion inspired the development of a precursor to cinematography in 1878, enabling full gait cycles of galloping horses to be captured for the first time (Muybridge 1887). From an evolutionary perspective, equid limbs have gone through an interesting sequence of anatomical changes, including limb elongation and reduction in the number of toes, which are thought to reflect selective pressures on locomotion (McHorse et al. 2019; Kaashoek et al. 2023). Modern horses are notable for many morphological adaptations that enable low energy costs and high speeds (Hoyt and Taylor 1981; Alexander and Jayes 1983; Hildebrand 1987). These include numerous structures that store elastic energy throughout the body (Dimery et al. 1986; van den Bogert et al. 1988; Biewener 1998; Minetti et al. 1999; Gellman and Bertram 2002), limb structures that can be seen as linkages (Hildebrand 1987; Usherwood 2022), running performance facilitated by (cursorial) limb proportions (Alexander and Jayes 1983; Hildebrand 1987), and high functional specialization in muscular architecture (Biewener 1998; Wilson et al. 2001; Brown et al. 2003; Payne et al. 2005a, 2005b).

Apart from fundamental insights into animal locomotion, numerous different approaches have been used to characterize equine locomotion for clinical applications. Kinematic asymmetry in the upper body is often quantified to assess equine lameness (Serra Bragança et al. 2018), and the practicality of modern kinematic measurement systems has resulted in increased popularity of using such systems in clinical practice. However, kinematics alone do not provide insight into internal structures and forces. These insights can be gained through direct measurements, although these typically require invasive (surgical) procedures (Biewener et al. 1983; van Weeren et al. 1992a; Faber et al. 2000; Haussler et al. 2001). A noninvasive alternative is to combine inverse approaches with a multibody-dynamic model of the (musculo)skeletal system (“musculoskeletal model”). Model-based inverse dynamics can provide unique insights into internal loads during locomotion (van den Bogert and Schamhardt 1993; Wilson et al. 2001; Bobbert and Santamaría 2005; Swanstrom et al. 2005; Zarucco et al. 2006; Bobbert et al. 2007; Pollock et al. 2008; Lim et al. 2015; Panagiotopoulou et al. 2016; Becker et al. 2019). However, most models that included the musculature have been of single limbs with the rest of the body highly simplified or completely omitted, and can therefore not be used for whole-body mechanics. A notable exception is Starke (2009), although their model's complexity prevented simulations of periodic gaits.

A drawback of inverse dynamic approaches is that they often cannot provide insight into why an animal moves in a certain way. A successful inverse dynamic simulation of a model will follow the measured movements of the animal nearly perfectly, and is thus limited to potentially idiosyncratic or pathological movements of the animal in question. Furthermore, inverse analyses can potentially obfuscate shortcomings in the model, depending on the research question and simulation approach (Modenese et al. 2013). Thus, inverse dynamics mostly preclude investigations into why an animal is moving in that manner in the first place.

Insights into gait selection can be acquired with predictive simulations—dynamic simulations where (a subset of) the joint motions are generated from first principles, without using kinematic or motion capture data as an input. Such simulations enable virtual experimentation—simulations of hypothetical changes to the musculoskeletal system, providing insights that cannot be acquired using physical experiments or inverse dynamics (van den Bogert and Schamhardt 1993; van Soest and Bobbert 1993; van Soest and Casius 2000; Swanstrom et al. 2005; Geijtenbeek et al. 2013; Sellers et al. 2013; Bishop et al. 2021; van Bijlert et al. 2021, 2024; Bianco et al. 2023). When actuating the model with muscles, movements are typically acquired using methods based on optimality principles: through optimization, neural inputs for the muscles are found that result in locomotion, while specific high-level objectives such as energetic expenditure or muscle activations are minimized (Ackermann and van den Bogert 2010; Srinivasan 2011; Geijtenbeek et al. 2013; Falisse et al. 2019). However, predictive simulation of locomotion is challenging, because both the modeled anatomy and the optimized control signals must be sufficiently accurate in order to produce realistic or meaningful simulations.

In a pioneering study, van den Bogert et al. (1989) presented torque-driven simulations of ponies. This model was later extended to include distal muscles and ligaments (van den Bogert 1989; van den Bogert and Schamhardt 1993). Although these simulations were not fully predictive, because some of the joints were controlled by prescribed kinematics, they represent the most comprehensive attempt at holistically modeling equine locomotion. Reductionist models of the horse used in predictive simulations have greatly increased our understanding of equine gait selection (Herr and McMahon 2000; Herr et al. 2002; Polet 2021), but to our knowledge, muscle-driven predictive simulations of whole body equine locomotion have never been published. Such simulations could have many applications, potentially providing insights on scaling effects in gait selection and evolution (Herr et al. 2002; McHorse et al. 2019), and could inform veterinary diagnostics (van den Bogert and Schamhardt 1993). An interesting fundamental application can be found by comparing the gaits of different horse breeds: for instance, Icelandic horses display a much broader locomotor repertoire than non-gaited horse breeds (Robilliard et al. 2007), and it has been suggested that mutations in the genes that encode spinal circuitry underly this phenomenon (Andersson et al. 2012). Testing this and related neuromechanical phenomena (Ijspeert and Daley 2023) would require a complex, muscle-driven model of the horse that is suitable for different control approaches.

**Fig. 1.**
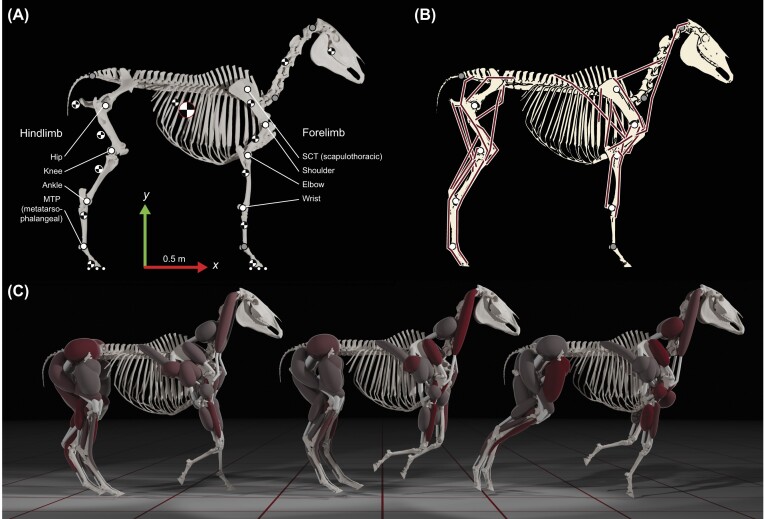
Overview of our model construction and simulation workflow. (A) Our model is based on a 3D (surface) scan of the skeleton. Model joint locations (large solid circles) are indicated. Named joints (white circles) are mobile, unnamed joints (gray circles) are immobilized for this study. The model has 5 contact spheres (small circles) per limb arranged in a horseshoe shape (only three per limb visible in this view, see Supplementary fig. S1). Center of mass locations (checkered circles) of the rigid bodies are indicated, the total body center of mass is enlarged for emphasis. Global axis directions are indicated with arrows (global *z*-axis is oriented towards the reader). (B) Overall joint and muscle topology of the model. Joints are the same as in panel (A). Muscle lines of action (thick lines), are projected over the skeleton in this view, although some muscles are on the medial side of the bones (see panel C). (C) Example of a muscle-driven, predictive simulation of our horse model during galloping. Control inputs for the muscles are found through optimization, and the resulting degree of muscle activation is visualized by color intensity (inactive muscles are light gray, active muscles are dark red). Relative muscle volumes are preserved in this visualization.

In this study, we describe the development of a new three-dimensional (3D) musculoskeletal model of the horse. To our knowledge, we present the first fully muscle-driven, predictive 2D simulations of horse locomotion (Fig. 1). Because little work has been done in this regard, the goal of this study is to investigate whether equine gaits and gait transitions can be recovered without including many of the anatomical intricacies that horses are known for. This sets a baseline that allows direct quantification of more complex horse models in future work, and also informs the expected performance of such models in comparative zoological and paleontological studies, where detailed anatomical data is either challenging to acquire or unavailable (Sellers et al. 2013; van Bijlert et al. 2021; Bishop et al. 2021). We have produced gait simulations with two different control strategies (1) using feedforward control, where control signals of the muscles are optimized in the absence of state feedback, and (2) using feedback control, where the control signals of the muscles are determined wholly by the kinematic states in the model. The feedforward-controlled simulations are intended to evaluate both the physical realism of the model during a variety of different gaits, including when tracking empirical joint kinematics, and to evaluate the overall performance of this control approach when modeling gait selection and transitions. The feedback-controlled simulations are intended as a proof of principle: feedback controllers have different model requirements, and in this study, we intend to demonstrate the flexibility of our model.

## Methods

### Overview

We have constructed a musculoskeletal model of the horse (*E. ferus caballus*). The modeling and simulation philosophy that guided this project was to go through a stepwise addition of model complexity. This is both practical, given that fewer muscles and joints simplify the locomotor control procedure, and also illuminating, because it provides insight into how different anatomical features contribute to gait dynamics. Thus, where previous models have focused on the anatomical intricacies of the distal limb (Riemersma et al. 1988; van den Bogert and Schamhardt 1993; Swanstrom et al. 2005), we have employed a more “top-down” approach, focusing on whole-body mechanics.

The skeleton and inertial properties of our model are based on the Dutch Warmblood breed. Muscle data specific to this breed were not available, and the literature on equine myology in general is relatively incomplete. As such, we aggregated muscles with similar actions into functional groups, resulting in 50 muscles in total. For this study, our goal was to explore equine locomotion and gait selection in a simplified model, but we have constructed the model with future additions and expansions in mind. The model has 21 joints, but several of these were immobilized for all the simulations in this manuscript (Fig. 1A, gray circles). The version of the model we simulated has three global degrees of freedom, and up to four degrees of freedom in each limb. Thus, it is a 3D model constrained to planar (2D) movements, with up to four hinge joints per limb (Fig. 1A, white circles). Most of the simulations we present had 17 degrees of freedom, with the following mobile joints: hip, knee, ankle, scapulothoracic (SCT), shoulder, elbow, and wrist (Fig. 1A). We also present simulations where we unlocked the metatarsophalangeal (MTP) joints. These simulations had 19 degrees of freedom. We never unlocked the metacarpophalangeal (MCP) joints.

We used this model for predictive simulations of gait using both feedforward and feedback-based control procedures. Using feedforward control we obtained a variety of gaits for our model over a wide range of speeds, and also produced one simulation where joint kinematics were tracked. Using feedback control, we present one acquired gait to demonstrate the flexibility of this model. We compared our simulations to empirical data from horses.

### Skeletal geometry and inertial parameters

We 3D-scanned a mounted horse skeleton at the Faculty of Veterinary Medicine, Utrecht University using an Artec Leo surface scanner (artec3d.com). The skeleton had no obvious abnormalities, and no further details regarding the provenance of the skeleton were available. Scans were preprocessed in Artec Studio 17, and then exported to Blender 3.1 (blender.org) for all further processing. The skeleton was placed in a natural, symmetric standing posture (Fig. 1A). To achieve perfect symmetry in the limb geometry, we reflected a single fore- and hindlimb across the sagittal plane.

3D inertial properties for our model were estimated by scaling the Dutch Warmblood horse dataset from Buchner et al. (1997) to our horse skeleton (see supplementary texts and Supplementary fig. S2 for details). The final body segment parameters are reported in Supplementary data file S1, sheet 1. Total body mass was 545 kg, which is nearly equivalent to the average in Dutch Warmblood horse in Buchner et al. (1997). Height at the withers was approximately 1.61 m, including a conservative ∼0.04 m to account for the absence of hooves, horseshoes, and soft tissue above the thoracic neural spines. Compared to the Dutch Warmblood horses studied in Bobbert and Santamaría (2005), our model is slightly smaller, but mass and withers height are both within two standard deviations of their trial means. Accounting for differences in segmental definitions, center of mass locations appear to be similar to thoroughbred racehorses (Kubo et al. 1992).

### Joints

The aggregate torso body has two translational (*x* and *y*) and one rotational (*z*) degrees of freedom with respect to the global reference system (Fig. 1A), and it is thus constrained to planar movements. This is mechanically equivalent to sliding on a plane. All the other joints in the model are hinge joints (Fig 1), with rotational axes orthogonal to the sagittal (*xy*) plane. We modeled SCT motion with a hinge placed at the level of the scapular spine, one fourth along the length of the scapula. The carpal and tarsal joints in the horse are compound joints with several distinct centers of rotation. We simplified these to simple hinge joints (wrist and ankle joint, respectively) based on comparisons with radiographs available from the “Imaging Anatomy” website, hosted by the University of Illinois (https://vetmed.illinois.edu/imaging_anatomy/index.html). The MTP joint was immobilized for all simulations, except for a sensitivity analysis. Our model includes more joints to facilitate future expansions of the model (such as an atlanto-occipital joint, Fig. 1). These were immobilized for the current study. Joint locations are reported in Supplementary data file S1, sheet 2. We do not explicitly model the mechanical effect of the sesamoids, the bone geometries are for visualization purposes only.

### Muscle and tendon properties

#### Muscle masses

To our knowledge, no complete muscle architecture dataset of equine locomotor muscles exists. For the hindlimb, Payne et al. (2005a) have presented a near-complete dataset. For the forelimb, we had to combine datasets from Brown et al. (2003), Payne et al. (2005b), and Watson and Wilson (2007). However, body-mass normalized muscles of several important shoulder and elbow muscles and three small hip-crossing muscles are not described in the equine literature. Muscle masses for 10 out of the 37 forelimb muscles, and 3 of the 36 hindlimb muscles were acquired from a different ungulate (*Rangifer tarandus*, reindeer), reported by Wareing et al. (2011). This accounts for 15.9% of the muscle mass in the forelimb, 2.1% in the hindlimb, and 7.8% of the total muscle mass in the model. For each locomotor muscle, we collated its mass (normalized to body mass). These data are summarized in Supplementary data file S1, sheets 3 and 4.

#### Functional groups

Muscle actions in the sagittal plane were determined using comparisons with the literature (Nickel et al. 1986; König and Bragulla 2007; Barone 2010; Budras et al. 2012), physical palpation in live horses, and comparisons with several equine plastinates at the Anatomy Department of the Faculty of Veterinary Sciences at Utrecht University. We eliminated redundant muscle functions based on their major contributions in the sagittal plane, and did not consider digital mobility. We also did not individually model muscle functions with a combined mass less than 0.5% of the limb's total muscle mass. The resulting 25 muscle functional groups per side are presented in Fig. 1.

When an anatomical muscle had multiple (posture-dependent) functions, we distributed its mass equally over several functional groups. Supplementary data file S1, sheet 5 shows the 25 functional groups (per side), which anatomical muscles contributed to their masses, and which anatomical muscle their lines of action were based on. When multiple muscles contributed to a functional group, we modeled the heaviest muscle. This procedure resulted in the hindlimb having an actuated MTP joint, even though digital flexion was not explicitly included when determining the 25 muscle functions. 3D muscle lines of action were reconstructed in Blender (Fig. 1B, Supplementary data file S1, sheet 6).

#### Contractile properties

We parametrized peak isometric force (*F*_max_, in *N*) of each muscle fiber using the following equation (Alexander 2006):


(1) \begin{eqnarray*}
{{F}_{{\mathrm{max}}}} = \frac{{{{m}_{{\mathrm{muscle\ }}}}\sigma }}{{\rho {\mathrm{\ }}{{L}_{{\mathrm{0\ }}}}}}\ \cos \alpha.
\end{eqnarray*}

Here, *m*_muscle_ is the mass of the muscle in question, *σ* is the specific tension of muscle tissue (0.3 MPa used here (Payne et al. 2005a, 2005b; Marx et al. 2006)), *ρ* is the density of muscle tissue (1060 kg m^−3^), *L*_0_ is the optimal fiber length (in m), and α is the pennation angle of the muscle fibers. Because the muscles in our model represent functional muscle groups, we set the pennation angle to zero, modeling all muscles as parallel-fibered. Muscle functional group masses follow from Supplementary data file S1, sheet 5. Thus, the only free parameter for each muscle is *L*_0_ (fiber length), and *F*_max_ then follows from equation (1).

Given the incomplete state of the equine muscle architecture literature, and because our model currently only incorporates functional groups, we tuned *L*_0_ and tendon lengths (*L*_T_, in meters) for our model using a method very similar to that outlined by Sellers et al. (2013), see supplementary texts for details. This method is based on the observation that fiber lengths (and implicitly, tendon lengths), are somewhat tuned to habitual locomotor joint ranges used by animals (Burkholder and Lieber 2001). We modified the implementation by Sellers et al., because fiber lengths of proximal muscles tend to be much longer than distal muscles (Brown et al. 2003; Payne et al. 2005a, 2005b). We manually tuned the muscle-tendon units to vary between 0.75 and 1.25 *L*_0_ (proximal) or 0.5 and 1.5 *L*_0_ (distal) when the joints are moved between a set of joint ranges (not accounting for tendon elasticity). This method self-corrects somewhat for small reconstruction errors in the muscle paths (Sellers et al. 2013), and ensures the model is capable of generating enough force at the desired joint ranges. However, this method ignores most of the functional specificity of the muscles. Many of the fiber lengths are within the reported ranges in the literature, but certain muscle groups required substantially altered fiber lengths to enable realistic gaits (see supplementary texts). The final contractile parameters for the model, compared to fiber lengths from the literature, are reported in Supplementary data file S1, sheet 5.

Maximal contraction speeds (*v*_max_, in *L*_0_ s^−1^) of horse muscle fibers have been reported in the literature (Rome et al. 1990; Marx et al. 2006; Butcher et al. 2010). There is a considerable spread in the measurements, even when accounting for temperature and fiber types, further complicated by between-muscle differences in fiber type composition (Kawai et al. 2009). Unless stated otherwise, we present simulations where we used 6.6 *L*_0_ s^−1^, representing a mixed fiber-type equine muscle (Supplementary data file S1, sheet 7). See supplementary texts for details on how we acquired this value, and two sensitivity analyses where we varied *v*_max_.

Activation dynamics, which model the fraction of Ca^2+^ bound to troponin within the myocyte (Hatze 1977; Winters and Stark 1988), can also affect stride frequencies in simulations (van Soest and Casius 2000). In our model, they are represented by the activation and deactivation time constants (τ_a_ and τ_d_, respectively; both in seconds). In particular, τ_d_ can be rate-limiting in locomotion (Marsh 1990; van Soest and Casius 2000). Unfortunately, the scaling between body size and muscle (de)activation dynamics is still unknown, and to our knowledge has never been measured in horses. However, an inverse relationship exists between τ_d_ and *v*_max_ (Close 1965), and *v*_max_ itself scales approximately with (body mass)^−0.125^ (Medler 2002). Based on this, we have opted to scale the time constants with (body mass)^0.125^. Assuming 70-kg body mass for the human-scale time constants in De Groote et al. (2016), we calculated a scale factor of 1.29 for the time constants in our 545-kg horse. Unless otherwise specified, our simulations used τ_a_ = 0.0194 s and τ_d_ = 0.0775 s for all the muscles.

We used the muscle model described by De Groote et al. (2016) for our feedforward simulations, and the model described by Millard et al. (2013) for our feedback simulations. Both models are standard, three-element Hill-type actuators (Hill 1938; Zajac 1989) (see supplementary texts).

### Optimization strategies

To acquire periodic locomotion in musculoskeletal models, we must find muscle activation patterns through optimization, often minimizing model outputs related to muscle activity or energy cost (Ackermann and van den Bogert 2010; Geijtenbeek et al. 2013; Falisse et al. 2019). The number of possible activation patterns is practically infinite, so finding suitable activation patterns is a computational challenge. We have performed gait simulations using both feedforward control, and feedback control. In feedforward control, muscle activation patterns are predetermined and do not change in response to the model states, so there is no state feedback. In pure feedback control, muscle activation patterns are wholly determined by changes in model states, and there is no predetermined muscle activation pattern. We implemented these diametrically opposed control strategies using simulation methods that are also very different in nature: in the feedforward-controlled simulations, we have used “direct collocation” (Ackermann and van den Bogert 2010; De Groote et al. 2016; Falisse et al. 2019; Dembia et al. 2020), and for the feedback-controlled simulations, we optimized the control parameters using a more traditional shooting method (Geijtenbeek et al. 2013).

In direct collocation, the entire trajectory (discretized into timepoints) is optimized alongside the muscle activation patterns. Dynamic consistency between the timepoints is enforced by constraints that can initially be ignored. Thus, the initial guess for the optimization can be a random trajectory that can initially ignore the laws of physics (e.g., a model floating forward at the desired speed (Falisse et al. 2019)). As the optimizer converges, constraint errors are reduced until physical laws are obeyed. In a shooting method, an initial guess for the activation patterns is used for many simultaneous simulations (i.e., numerical integrations over time that all obey physics). The movement trajectory itself is not directly modified—in each iteration of the optimizer, the muscle activation patterns are modified and the resulting movements are found by doing more simulations. This is an important difference when compared to direct collocation, where all the timepoints of the trajectory itself are altered simultaneously with each iteration of the optimizer (De Groote et al. 2016), and the trajectories are not acquired by numerical integration over time using an initial state. We will refer to converged trajectories from both modalities as simulations.

For our feedforward-controlled simulations, we broadly follow the same methodology described in detail in van Bijlert et al. (2024) (in review). Our approach using feedback-controlled simulations was an extension of the approach introduced in Geijtenbeek et al. (2013). We attempted to keep the models as consistent as possible between simulation methods. However, to implement direct collocation we used OpenSim Moco v4.4 (Seth et al. 2018; Dembia et al. 2020), and to implement feedback control we used SCONE and Hyfydy (Geijtenbeek 2019; 2021), and these software packages use slightly different implementations of a Hill-type muscle model (Hill 1938; Zajac 1989; Millard et al. 2013; De Groote et al. 2016). Since these differences are minor, we do not expect them to significantly affect the results, especially in comparison to the much larger difference in the control methodology itself. We will describe the two methodologies in the next sections, focusing on where we depart from the previous implementations. See supplementary texts for more details.

#### Feedforward-controlled simulations using direct collocation

Our overall approach was very similar to van Bijlert et al. (2024) (in review), with necessary modifications to extend the workflow to quadrupedal gaits and more complex models. Specifically, it proved infeasible to find realistic gait solutions using quasi-random guesses in the model with full complexity. We were able to find good solutions by initially simplifying the model: we immobilized the ankle, SCT, and wrist joints (using “WeldJoints” in OpenSim), removed bi- and tri-articular muscles unless it would result in an un-actuated joint, and increased *F*_max_ of the remaining muscles to compensate. We used converged solutions from simplified models as the initial guess for the model with full-complexity (restoring *F*_max_ to values found via equation (1)), resulting in a wide array of gaits found without using motion-captured kinematics. These simulations can be considered fully predictive. We have also performed one tracking simulation, where we included the squared deviations from empirical kinematics during the trot (Back et al. 1995a, 1995b) in the cost function (see supplementary texts). This tracking simulation is different from traditional inverse dynamics, because dynamic consistency is maintained by potentially allowing deviations from the empirical data, as discussed in Fox (2024).

We first found periodic walking gaits for the simplified model from quasi-random initial guesses—a dynamically inconsistent trajectory where the model floated forward at the target speed (see supplementary video, and code examples on our project page). The target speed for these simulations was 1.25 m s^−1^, because that is close to the optimal walking speed of ponies and horses (Hoyt and Taylor 1981; Minetti et al. 1999), but different stride lengths were explored by prescribing stride durations. These formed the initial guess for walking simulations in the more complex model. Converged solutions in the complex model were then used as the initial guess for optimizations with a sequentially higher target speed (in steps of 0.5 m s^−1^), allowing us to explore the speed-ranges and gait transitions. Our walking solutions spontaneously transitioned to either pacing or tölting using this procedure (see results), but did not transition to galloping. To find galloping gaits, we first searched for galloping using the simplified model (with higher target speeds). We then used an adequate solution as the initial guess for the more complex model, and then sequentially increased the target speed as above. We define the top speed of our model as the highest target speed at which the optimizations would still converge.

As is common when using this approach (Ackermann and van den Bogert 2010; Falisse et al. 2019), we initially found periodic gaits by simulating half-strides while enforcing bilateral (cross-)symmetry and periodicity of all the states. To find asymmetric gaits such as galloping, we transformed these half-strides into full strides, and enforced regular periodicity of the states for subsequent optimizations.

Our direct collocation optimizations used a multi-objective cost function (see supplementary texts for a full mathematical description). The main terms were (1) metabolic cost of transport (MCOT, in J kg^−1^ m^−1^), using the phenomenological model of Bhargava et al. (2004), (2) the integral of muscle excitations cubed, which we refer to as “fatigue,” following Ackermann and van den Bogert (2010). Fatigue has broader definitions in different neuromuscular contexts (Enoka and Duchateau 2016), but we adopt the narrow definition used by Ackermann and van den Bogert (2010) for the context of musculoskeletal simulations. Secondary costs with a much lower weight were included to improve numerical convergence. In the main manuscript, we only present results using MCOT as the main objective unless specified otherwise, but we performed a sensitivity analysis where we experimented with combinations of fatigue and MCOT, and only fatigue (see supplementary texts). We used OpenSim Moco v4.4 (Seth et al. 2018; Dembia et al. 2020) for our direct collocation optimizations. We provided code examples and all simulator outputs on our project page (see data availability).

#### Feedback-controlled simulations using optimized proprioceptive reflexes

Our feedback control strategy generates muscle excitation signals from delayed proprioceptive sensory signals, essentially modeling a rudimentary spinal reflex control network. The sensory signals consist of both muscle length and muscle force, representing signals from muscle spindles and Golgi tendon organs, respectively. The feedback signal *U* (i.e., the excitatory input) of each neuron was calculated as:


(2) \begin{eqnarray*}
U\ = {\mathrm{\ }}C_{0}\ + {\mathrm{\ }}k_{\mathrm{F}}\ \cdot \ F\ + \ k_{\mathrm{L}}\ \cdot \ L
\end{eqnarray*}

Here, *k*_F_ and *k*_L_ are the force and length feedback gains, respectively; *C*_0_ is the constant feedback offset; *F* and *L* are the delayed normalized muscle force and fiber lengths, respectively. *C*_0_, *k*_F_, and *k*_L_ were iteratively optimized based on our high-level gait objective.

Following the organization of the elementary proprioceptive pathways found in the mammalian spinal cord, we included both monosynaptic and antagonistic pathways for muscles sharing the same joint(s). Monosynaptic length reflexes were always excitatory, while antagonistic length reflexes were always inhibitory. Force reflexes were allowed to be both excitatory or inhibitory. Each pathway included a neural delay, based on the estimated length of the neural reflex pathway.

The model used in our feedback-controlled simulations also incorporates explicit joint limit torques (500 Nm rad^−1^), which are applied when joint rotations exceed a joint-specific range. These joint limit torques represent the forces naturally applied by ligaments and bony structures to prevent hyperflexion and hyperextension, and are required to prevent the model from collapsing during the simulation, which in turn can cause the optimizer to get stuck in local minima.

Our different gaits were generated by optimizing the feedback gains and offsets, minimizing MCOT (Bhargava et al. 2004). Both the feedback gains and offsets remained constant for the duration of each simulation. The feedback controller was implemented using SCONE (Geijtenbeek 2019), and the optimization was performed in SCONE using Covariance Matrix Adaptation Evolutionary Strategy (CMA-ES) (Hansen 2006; Geijtenbeek et al. 2013). We simulated the model using the Hyfydy simulation engine (Geijtenbeek 2021).

### Evaluation of gaits and dynamics

Because we are introducing a new model and workflow in this paper, we have focused on presenting broad comparisons between simulated gaits found using feedforward control, and empirical data from horses. We compared footfalls and ground reaction forces (GRFs) to empirical data from the literature (Merkens et al. 1986, 1993; Hildebrand 1989; Weishaupt et al. 2004; Witte et al. 2006; Self Davies et al. 2019), in order to evaluate individual gaits. We combined these with comparisons to empirical stride lengths and duty factors (Weishaupt et al. 2010), to evaluate gait selection across different target speeds. The trot has been recognized as a relevant gait to clinical decision making (Serra Bragança et al. 2018). Therefore, we also compared joint kinematics from our simulations to empirical data of trotting warmblood horses from (Back et al. 1995a, 1995b). We selected the trials they presented that were corrected for skin-displacement following van Weeren et al. (1992b). We compared the joint kinematics of both a fully predictive trial, and a trial where kinematics were added as a tracking term to the cost function (see supplementary texts). In this tracking simulation, we also compared muscle activations to surface electromyographic (EMG) data from Smit et al. (2024).

The EMG dataset was based on data from three horses during trot on a treadmill, ten strides each. Each signal was aligned to the initial contact of the limb that that the signal was collected from. The signals were rectified, enveloped, and resampled to 101 datapoints per stride, see Smit et al. (2024) for details. Because it is not possible to instruct horses to perform a maximal voluntary contraction, we normalized the EMG signal amplitudes to the median peak amplitude over all ten strides per horse. We plotted the average and standard deviation of all 30 strides. We compared these to muscle activations from the corresponding muscles (13 in total) of our model during a simulation of the trot where joint kinematics were tracked in the cost function. In our simulations, muscle activations were bounded between 0 and 1, with 1 representing maximal activation. Because the trot was a submaximal gait for our model, some of the muscles were not highly activated. To enable a more direct comparison to the EMG signals, simulated activations were plotted both without and with normalization to peak activation during the stride.

Empirical data were digitized using WebPlotDigitizer 4.0 (https://apps.automeris.io/wpd4/). We defined a stride as the distance traveled between successive contacts of the same limb. GRFs were reported as a fraction of total bodyweight (BW). We did not quantitatively compare muscular outputs (e.g., forces, metabolic costs) to measurements—due to our simplifications to the musculature, it is unlikely that such a comparison would be meaningful. However, MCOT was the main optimization criterion in most of our simulations, and we thus compared relative differences in MCOT to assess the optimality of the gaits we have found.

## Results

Although we have simulated our model at varying levels of complexity, unless otherwise specified, we present outputs from the model with mobile hip, knee, ankle, SCT, shoulder, elbow, and wrist joints. This model had 17 degrees of freedom and 50 muscles (17D50M). We will first highlight a selection of the gaits that we acquired using feedforward control. Next, we evaluate the extent to which this feedforward control method was able to model gait selection, and what factors affected this. Lastly, we present a simulation acquired using feedback control, to demonstrate the flexibility of the model. We have provided a video that demonstrates the gaits described in the manuscript, and an example of the simple to complex optimization sequence used to acquire the feedforward-controlled gaits (Supplementary Video S1).

### Gaits acquired using feedforward control

#### Walk

**Fig. 2.**
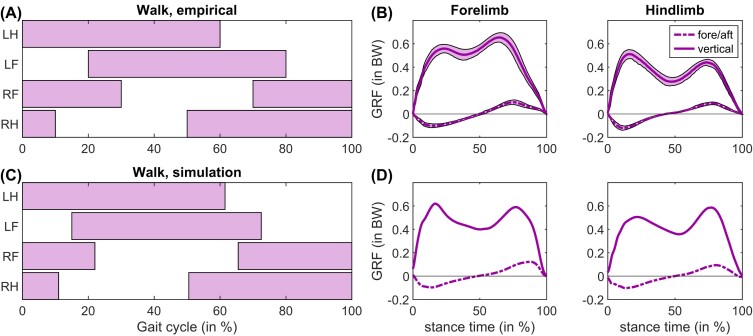
Empirically determined (A) footfalls and (B) GRFs (speed range 1.44–1.79 m s^–1^) during walking gait in horses (top row) compared to (C) footfalls and (D) GRF in our simulation model (bottom row, 1.25 m s^–1^). Footfalls were digitized from Hildebrand (1989), and the GRFs were digitized from Merkens et al. (1986).

Simplified models and the 17D50M model were both capable of walking gaits, showing M-shaped (double-humped) vertical GRFs. Fig. 2 shows an example of a walking solution using the 17D50M model. Compared to empirical data, the phase lag between forelimbs and hindlimbs tended to be lower in our model (∼5% in Fig 2.), although this was variable between different solutions. This can also be somewhat variable in horses, and some of our walking solutions could be classified as a “slow walking pace” (Hildebrand 1965).

#### Pace and trot

**Fig. 3.**
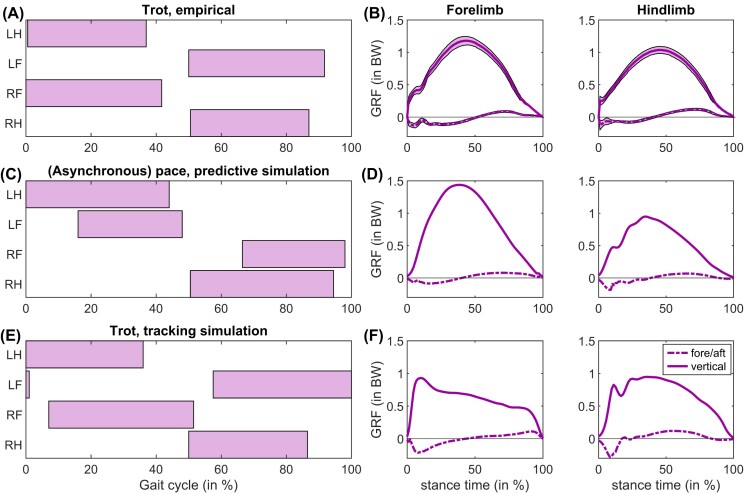
(A) Footfalls (3.5 m s^–1^) and (B) GRFs (speed range 3.9–4.3 m s^–1^) empirically determined in trotting horses, compared to (C, D) a fully predictive pacing gait (3.25 m s^–1^) and (E, F) a trotting gait (3 m s^–1^) acquired with a tracking cost added to the cost function. Footfalls were digitized from Weishaupt et al. (2004), and the GRFs were digitized from Merkens et al. (1993).

Our model is symmetric and constrained to planar (2D) movements, pacing and trotting are therefore equivalent gaits. In many of the predictive sequences (see next section), we found gaits that could be classified as a pace (Fig. 3C, D). The GRFs showed a reasonable correspondence to empirical data (Fig. 3B, D), although in a true pace, the forelimbs and hindlimbs strike nearly simultaneously (Hildebrand 1965). While some phase lag between fore and hindlimbs is not unusual (Hildebrand 1965; Rhodin et al. 2022), fore-hind asynchrony was more pronounced in our model.

In the pacing gait presented in Fig. 3C, D, stride length was 1.59 m, approximately 37% less than in warmblood horses at this speed (Weishaupt et al. 2010). As a result, peak joint excursions were much less pronounced when comparing joint kinematics to empirical data from Back et al. 1995a, 1995b) (Supplementary fig. S3). To evaluate whether the muscles in the model were physically capable of generating required joint torques over the joint ranges that horses adopt, we produced a simulation that included deviations from empirical joint kinematics as an extra tracking cost (see supplementary texts). This tracking simulation had greatly improved kinematics (Supplementary fig. S3), although from the footfalls it is evident that there was still some fore-hind asynchrony (Fig. 3E). The GRFs also showed a left-skew, reaching their peak value earlier in the stance phase than in the empirical data (Fig. 3F). This may be related to the immobilization of the distal joints in our model, limiting the spring-like leg behavior that is known to occur in horses (Biewener 1998; McHorse et al. 2019; Kaashoek et al. 2023).

Although we only tracked joint angle deviations in the tracking simulation, muscle activations in the simulation showed reasonable correspondence to empirical EMG signals, despite model simplifications (Supplementary fig. S4). In horses, some muscles show anticipatory activity to the limb initial contact (e.g., M. semitendinosus), which our simulation failed to capture. In some muscles, the duration of muscle activation predicted by our simulation was also shorter than the empirical data (e.g., M. triceps brachii caput longum).

#### Collected gallop

**Fig. 4.**
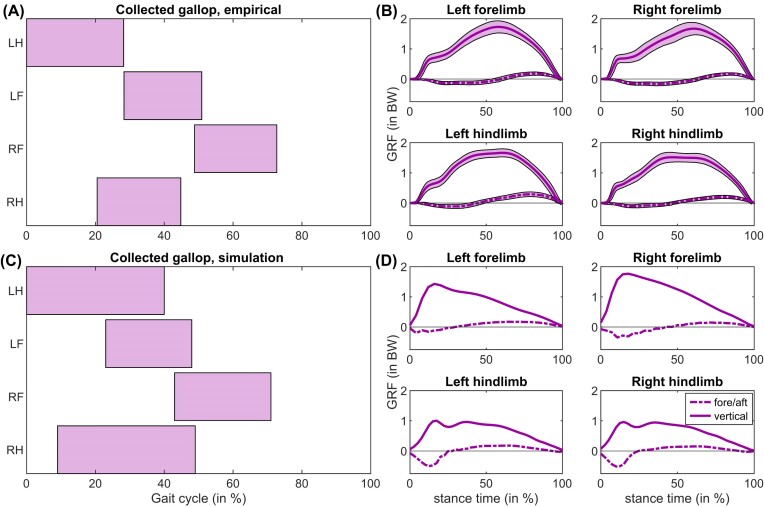
(A) Footfalls (at 9 m s^–1^) and (B) GRFs (speed range 9.1–13.7 m s^–1^) empirically determined in galloping horses compared to (C) footfalls and (D) GRFs in our model (8.75 m s^–1^). This simulation was acquired by raising *v*_max_ from 6.6 to 16 *L*_0_ s^–1^ (see supplementary texts), using MCOT as the main objective in the cost function. Footfalls were digitized from Witte et al. (2006), and the GRFs were digitized from Self Davies et al. (2019).

Horses typically adopt a transverse, collected gallop (Fig. 4A). Transverse (as opposed to rotary) signifies that the contralateral forelimb is the next leg to contact the ground after the second hindlimb contact. Collected (as opposed to extended) signifies that the limbs are close together during the flight phase (Hildebrand 1989; Bertram and Gutmann 2009). We present a transverse collected gallop (Fig. 4C), but the distinction between the transverse and rotary gallop, thought to be related to longitudinal stability, is not meaningful in our 2D-constrained simulations. During galloping at 8.75 m s^−1^, simulated footfalls showed a reasonably good correspondence to horses galloping near this speed (Fig. 4C), although there was a pronounced fore-hind asymmetry in duty factors (see next section). 8.75 m s^−1^ was the highest target speed at which a galloping simulation would converge using a *v*_max_ of 16 *L*_0_ s^−1^. Top speeds ranged between 7.75–9.75 m s^−1^, using values of *v*_max_ between 6.6–16 *L*_0_ s^−1^ and different cost function weightings (see Sensitivity Analysis 1 in the Supplementary Texts). Similar to the trot presented in Fig. 3F, GRFs during galloping showed a left-skew (Fig. 4D). At 8.75 m s^−1^, stride length was 2.81 m, ∼36% lower than expected for thoroughbred racehorses (Witte et al. 2006). Increasing the (de)activation time constants by a factor of two resulted in a stride length of 2.83 m (∼0.6% increase), so we concluded that our current gait solutions were not strongly affected by (de)activation dynamics.

#### Ambling gait (reverse tölt)

**Fig. 5.**
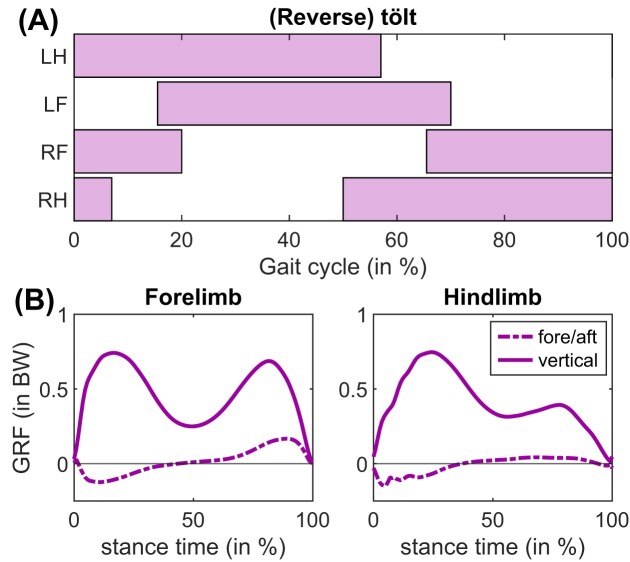
Panel (A) shows footfalls and panel (B) shows GRFs in our model during a transitional ambling gait we refer to as a “reverse tölt” at 2.25 m s^–1^. The forelimb shows walking dynamics (M-shaped GRF), whereas the hindlimb GRF shows a transitional state between walking running dynamics, recognizable by the attenuated second peak.

In certain optimization sequences, our model transitioned to an ambling gait at 2.25 m s^−1^ (Fig. 5). The M-shaped GRF in the forelimb clearly shows walking dynamics, signifying pendular or vaulting mechanics (compare Fig. 5B to Fig. 2B). We distinguish this from running, where a bell-shaped GRF signifies bouncing mechanics (Fig. 3B). In the ambling gait we found, the hindlimb was in a transitional state between walking and running, recognizable by the attenuated second peak in the GRF (Fig. 5B). This state resembles the transitional GRFs presented in Bishop et al. (2018). In Icelandic horses, transitional tölt gaits show walking dynamics in the hindlimbs instead of the forelimbs (Biknevicius et al. 2004), thus the gait we present in Fig. 5. may perhaps be called a reverse tölt. We are unaware of any studies describing this gait in horses, but based on limb-mechanics it can be inferred that elephants adopt this gait at intermediate speeds (Ren and Hutchinson 2008). At higher speeds, the hindlimbs adopted true running dynamics (bell-shaped GRF, such as in Fig. 3D), whereas the forelimb GRFs became transitional until the model transitioned to pacing at even higher speeds (see next section).

### Gait selection and transitions using feedforward control

#### Stride lengths

**Fig. 6.**
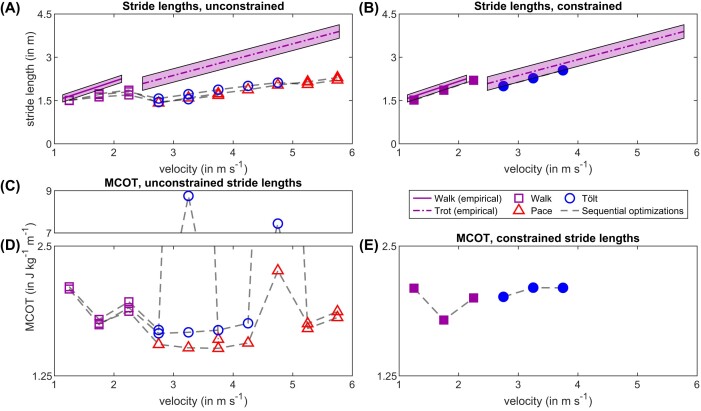
Stride lengths, MCOT, and gait transitions as a function of speed. Sequential optimizations are connected by dashed gray lines. All simulations minimized MCOT as the main cost and used a *v*_max_ of 6.6 *L*_0_ s^–1^. (A) In simulations with unconstrained stride lengths, stride lengths were underestimated when raising the target speed beyond 1.25 m s^–1^. The model transitioned between walking and running dynamics near the expected transition speed, switching either to a pace or a tölt (See Figs. 3 and 5). (B) When constraining stride lengths to fall within the standard deviations of empirical measurements, simulations converged towards the shortest stride lengths available. Symbols are filled to signify that the stride lengths were prescribed. (C) and (D) Without a constraint on the stride lengths, MCOT showed a local minimum at a walking speed of 1.75 m s^–1^, and a second minimum when pacing 3.75 m s^–1^. Transitioning to tölting instead of pacing was always costlier, and in both presented sequences culminated in an extremely costly local optimum before transitioning to a much more economical pacing gait. (E) When constraining stride lengths, MCOTs were similar to the unconstrained simulations at walking speeds, but substantially higher at running speeds, where the model adopted a tölting gait. This shows that empirically determined stride lengths were a local optimum for our model in the presented gaits. Empirical data from Weishaupt et al. (2010).

At walking speeds, stride lengths in our model were close to empirical stride lengths from Weishaupt et al. (2010), if MCOT was the main term in the cost function (Fig. 6A). When increasing the target speed beyond 1.25 m s^−1^, the stride lengths adopted by our model were lower than empirical measurements across the entire speed range, between 34 and 42% lower over a speed range of 2.75–5.75 m s^−1^ (Fig. 6A). Stride lengths and frequencies are inversely related, our model thus also adopted stride frequencies that were higher than empirical measurements. We performed a sensitivity analysis using alternative cost function formulations that penalized muscle fatigue (i.e., peak neural input, following (Ackermann and van den Bogert 2010), see supplementary texts), which resulted in even shorter strides (Supplementary fig. S5). Similarly, we investigated whether lowering *v*_max_ to 1.79 *L*_0_ s^−1^ would reduce stride frequencies and thus increase stride lengths (see Sensitivity Analysis 2, supplementary texts), but this also moderately reduced stride lengths (Supplementary figs. S6). We will elaborate on this issue in the discussion. We were able to circumvent this by prescribing the empirical stride length relationship from Weishaupt et al. (2010) in the controller, although this would complicate interpretations of gait selection (Fig. 6B). In the following paragraph, we will therefore focus on the unconstrained simulations presented in Fig. 6A.

#### Gait transitions

Horses and other mammals display a gait transition from walking to running (trotting, pacing, tölting, etc.) between Froude numbers (non-dimensional speed) of 0.4–0.6 (Hoyt and Taylor 1981; Alexander and Jayes 1983). Assuming a hip height of ∼1.11 m, this corresponds to a gait transition between 2.08 and 2.55 m s^−1^ for a horse similarly sized to our model. When increasing the target speed above 2.25 m s^−1^, our model spontaneously switched to a pacing or reverse-tölting gait (Fig. 6A, individual gaits presented in Figs. 2, 3, and 5). Around the walk to run transition, we also recovered locally optimal gaits (e.g., gaits with a two-to-one polyrhythm between hind and forelimbs), which occurred more frequently when the bounds on the states in the optimizer were too wide, but also when *v*_max_ was lowered (Supplementary fig. S6). Despite these complications, our model always transitioned from walking to running dynamics, usually pace or tölt, near the transition speed expected for horses of this size (Alexander and Jayes 1983).

Across the walk to pace transition, MCOT also showed a pattern qualitatively similar to empirical data from horses (Hoyt and Taylor 1981; Minetti et al. 1999): a U-shaped progression and minimum MCOT during walking, and a shallower, lower minimum during pacing (Fig. 6D). We caution the reader that these outputs should only be used to compare relative patterns of MCOT, as our aggregated muscle functional groups likely lead to underestimations of MCOT. Both tölting sequences in Fig. 6D had higher MCOT than pacing, culminating in extremely costly local optima (Fig. 6C), before transitioning to a much more economical pacing gait (Fig. 6D). All sequences used MCOT as the main objective in the cost function, the reverse tölt was thus a locally optimal gait in our simulations. Duty factors of these simulation sequences (Supplementary fig. S7) further support that tölting can be interpreted as maintaining walking dynamics across the walk to run transition speed. Compared to empirical data, our pacing simulations maintained higher duty factors in the hindlimbs, and lower duty factors in the forelimbs (Supplementary fig. S7), which can also be seen in the footfalls (Figs. 3C, 5C).

Cursorial mammals typically transition from symmetrical to asymmetrical running gaits at Froude numbers between 2 and 3 (Alexander and Jayes 1983), corresponding to a range of 4.67–5.72 m s^−1^ for our model. In simulations of full strides, our 17D50M model did not transition to galloping when sequentially increasing the target speed of a pacing solution. We were able to find this gait in our model by increasing the target speed in a simplified model. We selected suitable galloping solutions based on the footfalls and used these as the initial guess for the complex model. Using a *v*_max_ of 6.6 *L*_0_ s^−1^, galloping was costlier than pacing for nearly all speeds considered (Supplementary fig. S8), and the fastest recovered pacing speed was higher than galloping (8.25 m s^−1^ versus 7.75 m s^−1^, respectively). This suggests that galloping was also a locally optimal gait in our model (see discussion). Interestingly, even though the progression in stride lengths was smooth, MCOT tended to show large spikes near the expected symmetric to asymmetric gait transition speed range (Supplementary fig. S8).

#### Effect of unlocking the MTP joints

**Fig. 7.**
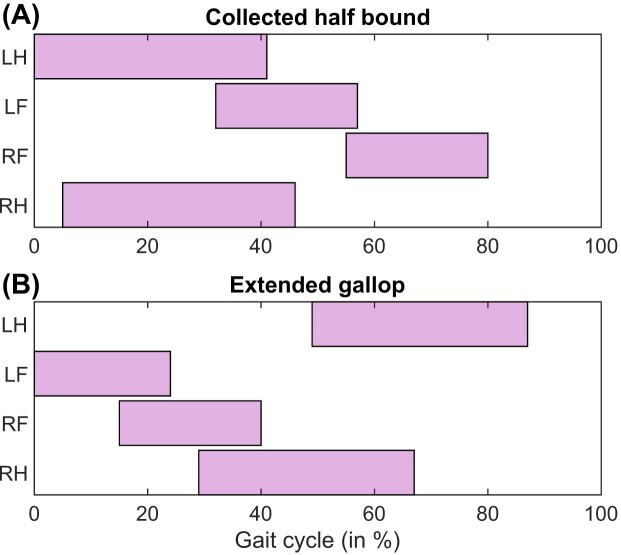
Footfalls during (A) collected half bound (6.75 m s^–1^) and (B) extended (rotary) galloping (5.25 m s^–1^) simulations. These gaits are not typically used by horses, although they are seen in other mammals.

Even though we did not set out to model digital flexion and extension, our procedure for determining functional groups resulted in actuated MTP joints (see methods). We therefore performed simulations where we enabled MTP mobility in the model (19D50M), to investigate whether this affected stride lengths and gait selection. At 1.25 m s^−1^, simulated stride length was within one standard deviation of the empirical data, ∼4.2% lower than the mean. However, at higher speeds the progression in stride lengths was mostly indistinguishable from the simulations with locked MTP joints (Supplementary fig. S9). When using galloping gaits as an initial guess, we found two other asymmetric gaits (Fig. 7): a half bound, where the hindlimbs are nearly symmetric but the forelimbs are not, and an extended gallop, where the limbs are extended during the flight phase. To our knowledge, horses do not normally adopt these gaits, although cheetahs and deer are known to use extended gallops, and jackrabbits use half bounds (Hildebrand 1989; Bertram and Gutmann 2009). Unlocking the MTP joint did not substantially alter stride lengths in our galloping simulations (Supplementary fig. S8).

### Feedback-controlled gait—Tölt

**Fig. 8.**
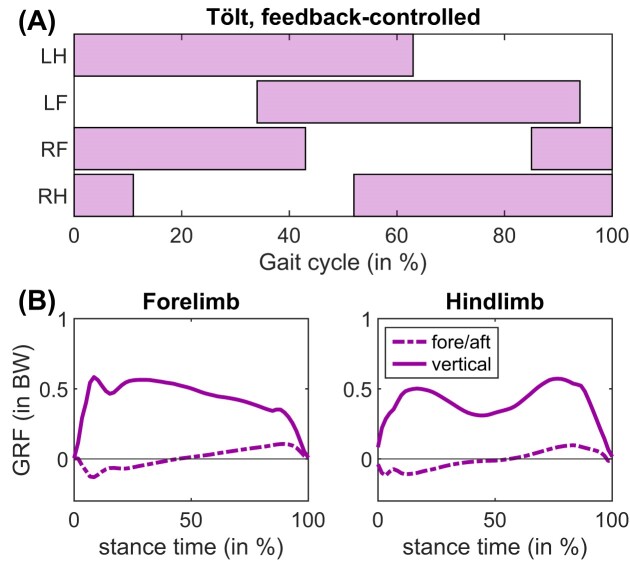
(A) Footfalls and (B) GRF in our model during tölt at 1.53  m  s^–1^, found using a spinal feedback-controller. The hindlimb shows walking dynamics (M-shaped GRF), whereas the forelimb shows transitional running dynamics (second GRF peak is attenuated, but not yet a true bell curve that is seen at higher speeds). We plot the average over 16 strides. Stride length was 1.61 m.

In our feedback-controlled simulations, the model adopted a transitional tölt that is seen in Icelandic horses (and certain other horse breeds; Biknevicius et al. 2004). It is characterized by running mechanics in the forelimbs, contrasted by walking mechanics in the hindlimb (Fig. 8, compare to Fig. 5). At a speed of 1.53 m s^−1^, stride length was 1.61 m, which is ∼12.5% lower than empirically determined in warmblood horses (Weishaupt et al. 2010).

## Discussion

### Gaits and gait transitions

Our model displayed a number of gait characteristics resembling those of real horses. In particular, GRFs broadly follow empirical patterns in simulations where the model footfalls match those of an existing gait (Figs. 2, 3, and 4). Even in simulated gaits typically not seen in horses (Fig. 7), the footfalls show reasonable agreement with other mammals (Hildebrand 1989). Our tracking simulation demonstrated that the model is capable of joint motions near to those empirically determined in horses (Supplementary fig. S3). Even though we only tracked joint kinematics in this simulation, muscle activation patterns roughly follow empirical EMG patterns, although the feedforward control strategy did not predict anticipatory muscle activity (Supplementary fig. S4). Thus, despite anatomical simplifications, our model can provide meaningful insights into limb dynamics and coordination. This is encouraging from the perspective of evolutionary biomechanics where soft-tissue data are often limited (Sellers et al. 2013; van Bijlert et al. 2021; Bishop et al. 2021), with the caveat that it may be required to investigate multiple prescribed stride lengths for a given speed.

Given that pacing and trotting are equivalent when using a symmetric model with a planar constraint, our simulations correctly predicted a gait transition (Fig. 6A) at the empirically determined transition speed (Alexander and Jayes 1983). These transitions occurred spontaneously, while keeping the cost function the same. The U-shaped progressions of the MCOT curves suggest pendular savings during walking, and a second optimum during pacing (Fig. 6D). The tölt was a locally optimal gait in our simulations: tölting had higher MCOT than pacing (nearly sixfold higher at 3.25 m s^−1^, Fig. 6C, D). The tölt is a four-beat run, and a collisional work perspective would predict lower costs than for pacing, a two-beat run (Ruina et al. 2005). Recently, four-beat tölting has been shown to be costlier than two-beat running (pace or trot) in animals that have a relatively low whole-body moment of inertia in the sagittal plane, including horses (Polet 2021). Although MCOT of tölting horses has not been directly measured, it has also been estimated to be higher than trotting based on GRF progressions (Waldern et al. 2015). Given the tölt's status as a costlier, locally optimal gait, it is therefore somewhat surprising that we recovered tölt-like gaits several times using both feedforward and feedback control (Figs. 5, 6, 8). The tölt (Biknevicius et al. 2004; Robilliard et al. 2007) has been suggested to only be part of the equine locomotor repertoire if a genetic mutation is present that codes for specific spinal circuitry (Andersson et al. 2012). Overall, we believe the neuromechanical intricacies of the tölt warrant further research.

At high speeds, cursorial mammals transition from symmetric to asymmetric gaits (Alexander and Jayes 1983), often attributed to MCOT minimization (Hoyt and Taylor 1981) or avoidance of peak musculoskeletal forces (Farley and Taylor 1991). We did not find this second gait transition when simulating our complex model. We have presented simulation results with cost functions that minimized MCOT, but we also did not recover this transition when we minimized peak excitations (which we refer to as “fatigue”), a cost-function that indirectly minimizes peak muscle forces (Ackermann and van den Bogert 2010; Srinivasan 2011). We only found galloping by first searching for suitable solutions in a simplified model. Keeping cost functions and musculotendon parameters constant, galloping had slightly higher MCOT than pacing in our simulations (Supplementary fig. S8). This may be due to the absence of spinal flexion in our model. In rigid-backed models with telescopic limb actuators, trotting and galloping have similar costs and top speeds (Polet and Bertram 2019), but elastic storage in the spinal structures increases galloping economy (Alexander et al. 1985). However, the equine literature is equivocal in this regard: galloping is more economical than trotting in standardbred horses (Minetti et al. 1999), but not in ponies (Hoyt and Taylor 1981). Our model's fastest galloping speed is likely also limited by the lack of spinal flexion: equine lumbo-thoracic and cervical mobility (Faber et al. 2000; Haussler et al. 2001; Gellman and Bertram 2002) and associated energy-storing structures (e.g., the nuchal ligament) may be required for modeling true maximal effort galloping, as demonstrated in simpler quadrupedal models (Yesilevskiy et al. 2018). Substantial simplifications to the distal limb anatomy in our model likely also limited top speed (discussed in the next section).

Not recovering a pace to gallop transition in our complex model may also be a limitation of the gradient-based optimizer used in the feedforward control strategy. The cost landscape in high-speed locomotion has many local optima, all with similar costs (Polet and Bertram 2019), which likely explains why we recovered many local optima across the trot-gallop transition speed (Fig. 6C, Supplementary fig. S8). Symmetric and asymmetric leg coordinations may represent two optima that cannot easily be bridged using a gradient-based optimizer, especially in complex models where the search space is large. Transitional states between the two gaits could have higher costs, preventing gradient optimizers from finding such a transition. A related computational phenomenon is discussed in more depth elsewhere (van Bijlert et al. 2024) (in review).

### Gait selection and model simplifications

From the perspective of modeling gait selection, the major limitation of the feedforward-controlled simulations was that at speeds higher than 1.25 m s^−1^, simulated stride lengths were shorter than empirically determined in horses, unless stride lengths were explicitly constrained (Fig. 6A, B, a discrepancy of 34%–42% over the selected range). When comparing MCOT between the unconstrained and constrained simulations (Fig. 6D, E), it is clear that the empirically determined, longer stride lengths are local optima for our model. This can also be observed in our feedback-controlled simulations (Fig. 8). Thus, while the simulations we have presented are both fully muscle-driven and predictive, the model and control strategies require further refinements before they can confidently be used for rigorous virtual experiments regarding gait selection of horses. We will discuss three possible reasons for this, and how we have tried to eliminate those possibilities.

#### Musculotendon parameters

A possible cause for the short stride lengths in our model is our approach for determining fiber and tendon slack lengths. Musculoskeletal simulations are very sensitive to the musculotendon parameters (Charles et al. 2022), and these parameters often require some tuning so that models can generate adequate forces over joint ranges relevant to locomotion (Sellers et al. 2013). Directly using all the empirical fiber lengths (including pennation) resulted in a poorly tuned model that could only walk with deeply flexed limbs. We considered this a failed simulation, and therefore do not present it. Presenting a universal tuning method that works for all models is an ambitious goal that we do not attempt here. Instead, we found joint tuning angles through experimentation. The resulting fiber lengths show a reasonable correspondence with empirical fiber lengths (see supplementary texts and Supplementary data file S1, sheet 5), but we made no attempt at modeling the high architectural complexity of equine muscles (Brown et al. 2003; Payne et al. 2005a, 2005b; Watson and Wilson 2007). Adding empirically determined joint kinematics as an extra tracking cost during simulations demonstrated that the muscles could generate the requisite forces when using wide joint excursions (Supplementary fig. S3). However, this was not the optimal gait for our model—MCOT was 2–3x as high as the pacing gaits in Fig. 7D, although a direct comparison is impossible due to the additional terms in the cost function.

Combining nearly 75 muscles into the 25 functional groups in our model represents a substantial simplification, and there is considerable scope to increase anatomical accuracy by defining additional muscle groups. However, the state of the equine musculature literature currently precludes this, since a complete dataset is not available even if inter-breed differences are ignored. Acquiring a complete equine dataset would likely improve our model estimates. However, while measuring muscle masses is relatively straightforward, other architectural measurements are more challenging to perform accurately: fascicle length measurements are very time-sensitive due to the onset of rigor (Burkholder and Lieber 2001), drying of the tendons affect the resting lengths (Galton and Shepherd 2012), and these are complicated by the logistical difficulties involved in dissecting a >500 kg animal. Accurate assessments of the internal tendon lengths would have to be included, and even if the relative lengths of individual fibers are measured via determination of sarcomerial overlap (Burkholder and Lieber 2001), the sample size would have to be impractically large to account for the large distribution of absolute fiber lengths within a muscle (Charles et al. 2022). Modeling the highly specialized equine musculature architecture may provide further challenges: representing 3D muscles as simple path point Hill-actuators tends to overestimate fiber excursions (Blemker and Delp 2006), and large deviations from architectural measurements are sometimes required to accurately model human joint-angle moment relationships (van den Bogert et al. 1998).

We are unaware of deactivation time constants ever having been measured in a horse, and given the nearly twofold variation in reported *v*_max_ of horse muscles (Rome et al. 1990; Marx et al. 2006; Butcher et al. 2010), even these data were not ideal inputs. However, top galloping speed in our model (8.75 m s^−1^ in the main manuscript) could also be achieved with twofold longer time constants than the base model (with only a 0.6% increase in stride length), so we consider it unlikely that these were affecting our simulations.

#### Model topology

We have constrained our model to move across the sagittal plane. Full-3D simulations are feasible with this model, but unlikely to add meaningful information because we have modeled all joints as hinge joints perpendicular to the plane of motion. This seems to be a reasonable simplification, since mediolateral GRFs are insubstantial across the entire equine speed range (Merkens et al. 1986, 1993; Self Davies et al. 2019). We also opted not to model scapular translations: to our knowledge, soft-tissue constraints on (equine) scapular motion have never been measured, and simplifying scapular motion to a pure rotation provided acceptable results. It seems unlikely that these simplifications limited the stride lengths, since the model is clearly able to adopt longer strides and joint excursions close to empirical data (Fig. 6, Supplementary fig. S3).

In contrast, lack of digital flexion in our model could have limited stride lengths. Especially at higher speeds, horses rely on elastic energy storage in numerous distal tendons and ligaments (Dimery et al. 1986; Biewener 1998), but this has also been demonstrated at low speeds (Riemersma et al. 1988; van Weeren et al. 1992a). However, unlocking the MTP joints did not result in substantially longer stride lengths in our simulations (Supplementary figs. S8, S9).

Horses also have several ligamentous anatomical features that we did not model. These include the “stay apparatus” (a patellar locking mechanism to maintain unilateral knee extension), the “reciprocal apparatus” (ligamentous coupling between the knee and ankle angles), the lacertus fibrosus (a ligamentous band that extends from the insertion of the biceps to cross the carpal joint, aiding wrist extension), the suspensory ligaments (which resist hyperextension of the MCP and MTP joints, and aid swing-phase flexion), and the check ligaments (ligaments that attach directly onto the digital flexor tendons) (Riemersma et al. 1988; van Weeren et al. 1992a; van den Bogert and Schamhardt 1993; Biewener 1998; Wilson et al. 2001; Swanstrom et al. 2005; Watson and Wilson 2007; Budras et al. 2012; Usherwood 2022). Thus, our model currently does not include any passive-coupling or linkages that could have enabled low/no muscle-work gait solutions (Usherwood 2022), or could have simplified control. Inclusion of these passive structures, including the spinal structures previously discussed, could result in longer strides becoming the optimal solution. They would likely also reduce energy costs and enable higher top speeds through more effective power transfer and energy storage (Biewener 1998), and improve the swing-phase behavior of our model. However, modeling these structures would increase the number of parameters that require tuning, and some structures would require technical additions to existing musculoskeletal simulators (e.g., accurately modeling the check ligaments would require the addition of branching tendons (Fu et al. 2024)). The work here sets a baseline, against which future additions to our model can be compared.

#### Simulation approach

Gait selection is a complex phenomenon that is not fully understood (Ijspeert and Daley 2023), and it remains unclear what cost functions are most appropriate in simulations (Ackermann and van den Bogert 2010; Srinivasan 2011; McDonald et al. 2022; van Bijlert et al. 2024). Unlike in human simulations (Ackermann and van den Bogert 2010), MCOT as the main cost outperformed a fatigue-based cost function in our simulations depending on the outputs that are deemed relevant. This is why we only presented MCOT-minimizing simulations in the main manuscript (with the exception of the tracking simulation). Stride lengths at walking speed were closer to empirical data (Fig. 6A), and we found the highest speeds for our model using MCOT as the main cost. Fatigue minimization resulted in substantially shorter strides, despite also normalizing by the distance traveled (Supplementary fig. S5). Peak musculoskeletal forces are thought to trigger gait transitions in horses (Farley and Taylor 1991), but fatigue-minimization, which also minimizes peak muscle forces (Srinivasan 2011), did not have this effect in our simulations. However, we found that inclusion of the fatigue term in the cost function was necessary to increase force-sharing amongst the muscles in our tracking simulation (Supplementary figs. S3, S4), as has been demonstrated in human simulations (Ackermann and van den Bogert 2010).

Short strides in our simulations may also have been an unintended side-effect of scaling massless Hill-type muscle model (Hill 1938; Zajac 1989). Scale effects on muscle functioning place unequal demands on the locomotor performance of small and large animals, although different accountings for the mechanisms exist (Scholz et al. 2006; Usherwood and Gladman 2020; Labonte et al. 2024). Many of these effects are accounted for in our simulations, such as effects of gearing and the “parasitic” work performed by gravity (Scholz et al. 2006; Labonte et al. 2024). However, there is an intriguing possibility that modeling muscles as massless actuators, as is currently standard practice (Soest and Bobbert 1993; van den Bogert et al. 1998; Ackermann and van den Bogert 2010; Sellers et al. 2013; Bishop et al. 2021; van Bijlert et al. 2024), unduly overestimates the contractile performance of animals with large muscles (Günther et al. 2012; Ross et al. 2018; Labonte et al. 2024). In an animal as large as a horse, we may thus be underestimating the costs of high contractile frequencies and shorter strides.

Simulated uncertainty (e.g., perturbations) can affect the optimal muscle activation patterns (Koelewijn and van den Bogert 2022). Lack of simulated uncertainty may provide an explanation for why we did not predict any anticipatory firing (Supplementary fig. S4). A collisional work perspective (Ruina et al. 2005) could suggest that short strides were the result of ground contacts that were too dissipative—but both increasing and decreasing the dissipation and friction parameters in the contact model did not appreciably affect stride lengths in our direct collocation experiments. Although we have searched for many different optima, a factor that is always challenging to rule out is that we simply did not try sufficiently varied initial guesses to find optima with longer stride lengths. One strategy that was unsuccessful in finding gaits with longer stride lengths was to use solutions with prescribed stride lengths (e.g., Fig. 6B) as the initial guess for unconstrained simulations—these always reverted to short strides, both in pacing and galloping simulations.

### Applications of our model

Because we intend to use our model for fundamental gait research, we have focused on its predictive capabilities. Since there are no gait measurements directly being tracked, predictive simulations generally require very sophisticated control approaches, and are very sensitive to tuning issues. These hurdles can be avoided to some extent in inverse dynamics or tracking simulations, demonstrated by the improved kinematics when adding a tracking term to the cost function (Supplementary fig. S3). Unfortunately, unlike in human simulations (Haralabidis et al. 2021), in animal simulations we cannot rely on joint dynamometry to improve musculotendon parameters. Nevertheless, there is considerable scope to add tracking terms such as 3D kinematics, GRFs, activations derived from EMGs (Dembia et al. 2020; Haralabidis et al. 2021; Fox 2024), and such analyses may inform further additions and refinements to the model. Although this was not the primary goal of the current study, our model can thus potentially be a very useful tool in veterinary diagnostics, as clinicians often have access to kinematics and GRFs (Serra Bragança et al. 2018). High-fidelity models have already been used to simulate the effects of certain pathologies in equine locomotion (van den Bogert and Schamhardt 1993; Swanstrom et al. 2005). Combined with the predictive, optimal control framework applied here, in future work it may be feasible to simulate compensatory whole-body movements in response to locomotor pathologies. More generally, we believe realistic physics simulations can play a role in reducing animal use, as a teaching tool and when generating visual effects for the movie and video game industries.

To aid these and other applications, we have made our model and simulation scripts freely available in multiple formats on our project page, including an extensible code template that performs the presented tracking simulation. To increase adaptability and extensibility, our model includes more joints than we used for the simulations in this project. This includes MCP, neck, and atlanto-occipital joints (Fig. 1). Future work could thus be focused on investigating the mechanical effects of neck motions, and passively coupled motions in the distal limb.

## Conclusion

We have introduced a new musculoskeletal model of the horse that we have made open-source. To our knowledge, we have presented the first predictive, fully muscle-driven simulations of equine locomotion. To set a baseline for future work, the goal of this study was to simulate a horse model that does not include many of the anatomical specializations known in horse distal limbs. Despite these simplifications, we were able to capture many of the general features present in the gaits of horses and other mammals, including realistic footfalls, ground reaction forces, and one of the two major gait transitions. In a tracking simulation, the model adopted joint angle deviations close to those empirically determined in horses. These findings demonstrate the potential of the model developed here, both from a fundamental but also a clinical perspective. However, unless explicitly prescribed in the controller, stride lengths in our simulations were substantially shorter than empirically determined, suggesting that we are currently not able to accurately model all aspects of gait selection. Considerable improvements could be made to the musculotendon parameters of the model once a complete dataset becomes available, and the inclusion of additional joints and ligaments may be required to find a spontaneous trot to gallop transition. Despite these limitations, we have successfully produced both feedforward and feedback-controlled simulations of horse gaits, which will form the basis of future neuromechanical simulation studies.

## Supplementary Material

icae095_Supplemental_Files

## Data Availability

We have constructed a project page on SimTK (a biomechanics repository): https://simtk.org/projects/shadowfax. SHADOWFAX stands for Simulated Horse Anatomy Demonstrating Optimal Walking & Fast ACCeleration. Our models, simulator outputs, and example scripts are reposited on this project page.

## Notes

From the symposium “Computational and Physical Models in Research and Teaching to Explore form-function Relationships” presented at the annual meeting of the Society for Integrative and Comparative Biology, January 2–6, 2024 in Seattle, Washington.
