# Supplementary material for: Muscle-Driven Predictive Physics Simulations of Quadrupedal Locomotion in the Horse
Source: Integr Comp Biol. 2024 Jul 13;64(3):694–714. doi: 10.1093/icb/icae095 (PMC11428545; doi:10.1093/icb/icae095)
Supplement: icae095_Supplemental_Files [file icae095_supplemental_files.zip › Supplementary Figures.pdf]

Supplementary Figures

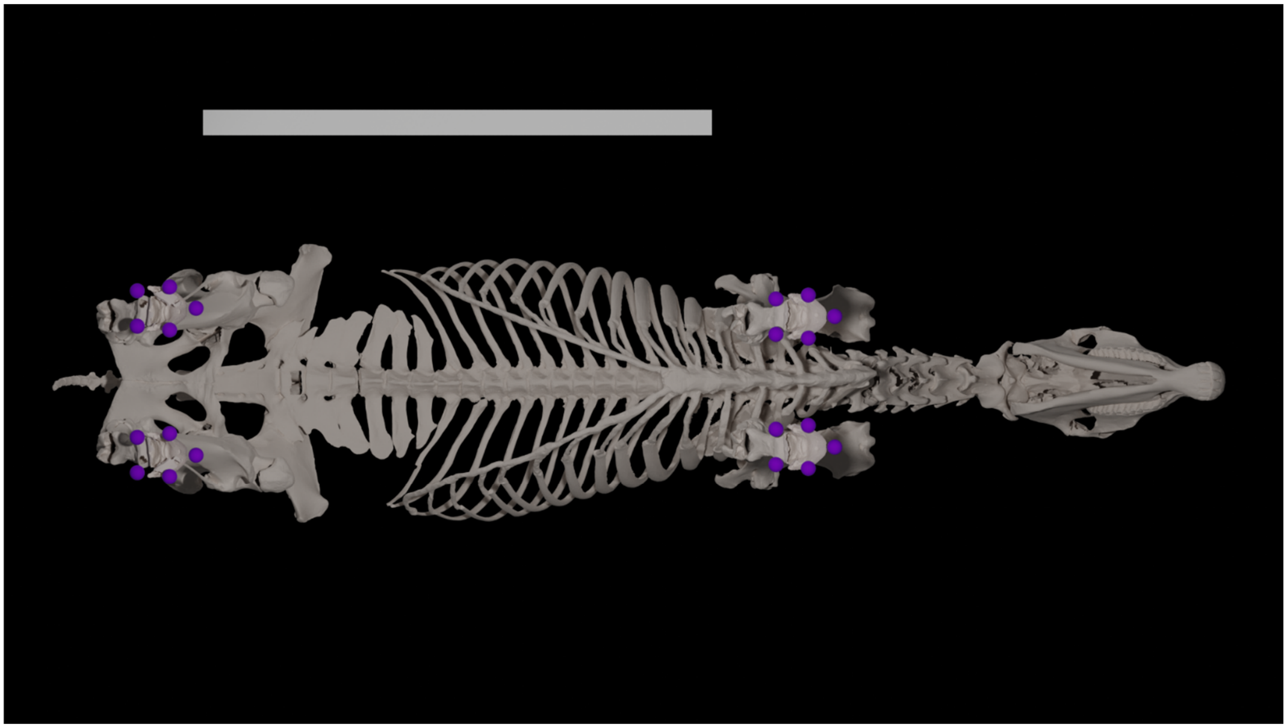

Supplementary Fig. S1. Orthographic projection of the horse model in ventral view, showing the contact sphere placement arranged in a horseshoe shape, following equine radiographs. The contact sphere radius was 0.015 m. The scale bar is 1 m.

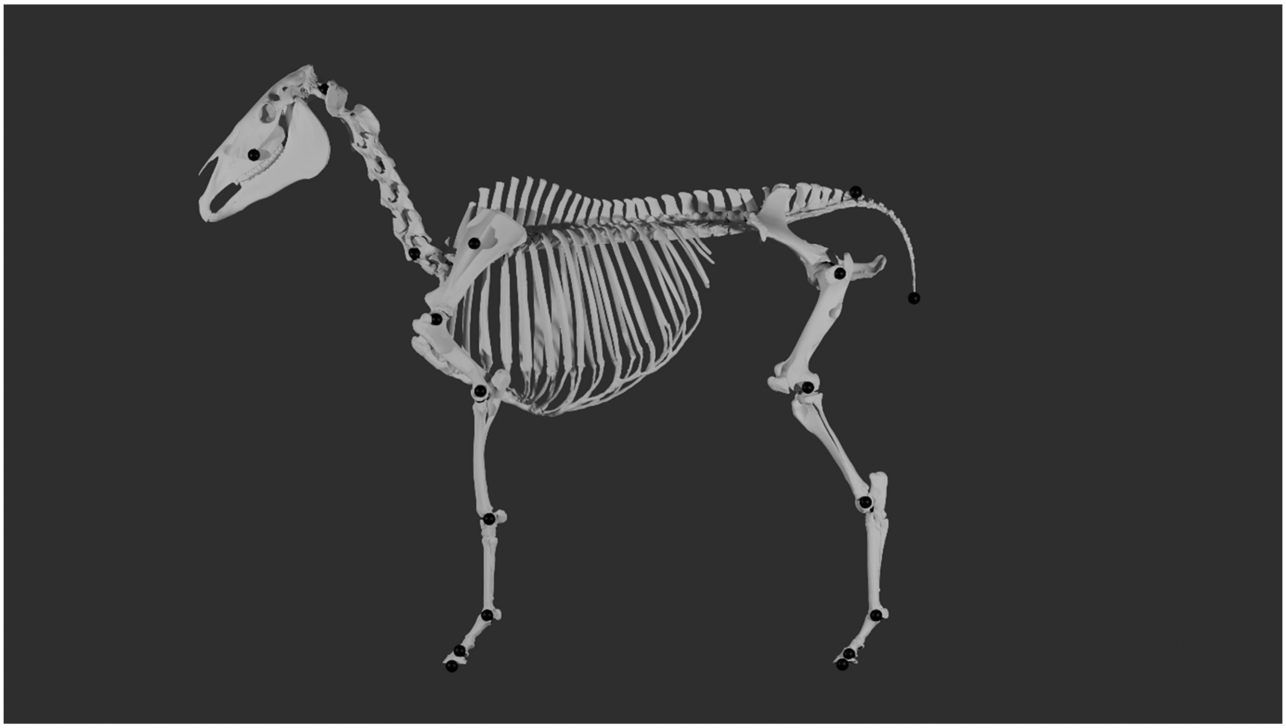

Supplementary Fig. S2. Marker placement on our horse skeleton following Buchner et al. (1993), to estimate the inertial properties of the rigid bodies. Resulting center of mass locations are visualized in Fig. 1 (main manuscript).

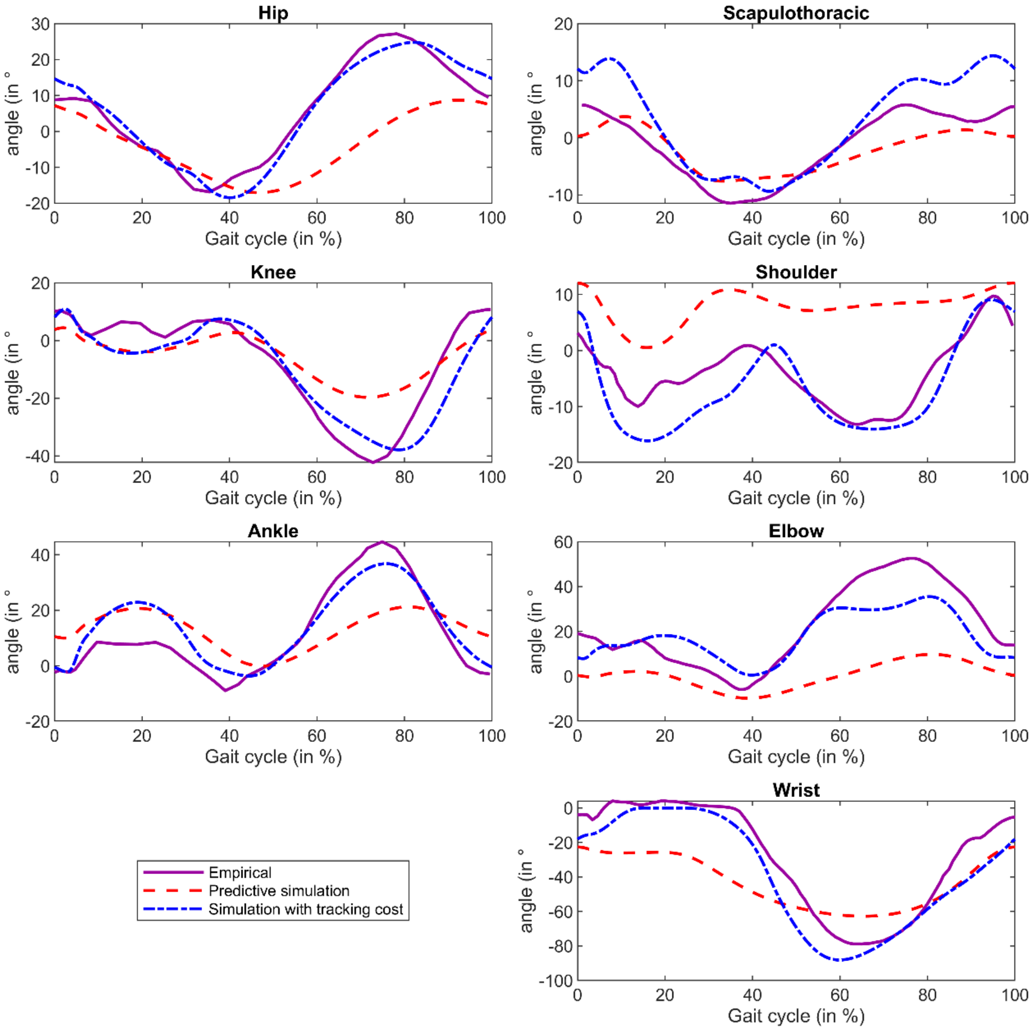

Supplementary Figure S3. Empirical joint angles of trotting horses (3 m s^{-1}), compared to joint angles acquired from a fully predictive simulation of the pace (3.25 m s^{-1}), and a tracking simulation of the trot (3 m s^{-1}) which included squared joint angle deviations in the cost function (see supplementary texts). All sequences are normalized to the initial contact of the respective limb, enabling cross comparisons of pacing and trotting. The predictive simulation had much smaller joint angle excursions. The joint angles were greatly improved in the tracking simulation, although fore-hind footfall timings were still asynchronous (Fig 3E, main manuscript). The empirical data are from Back et al. (1995a; 1995b).

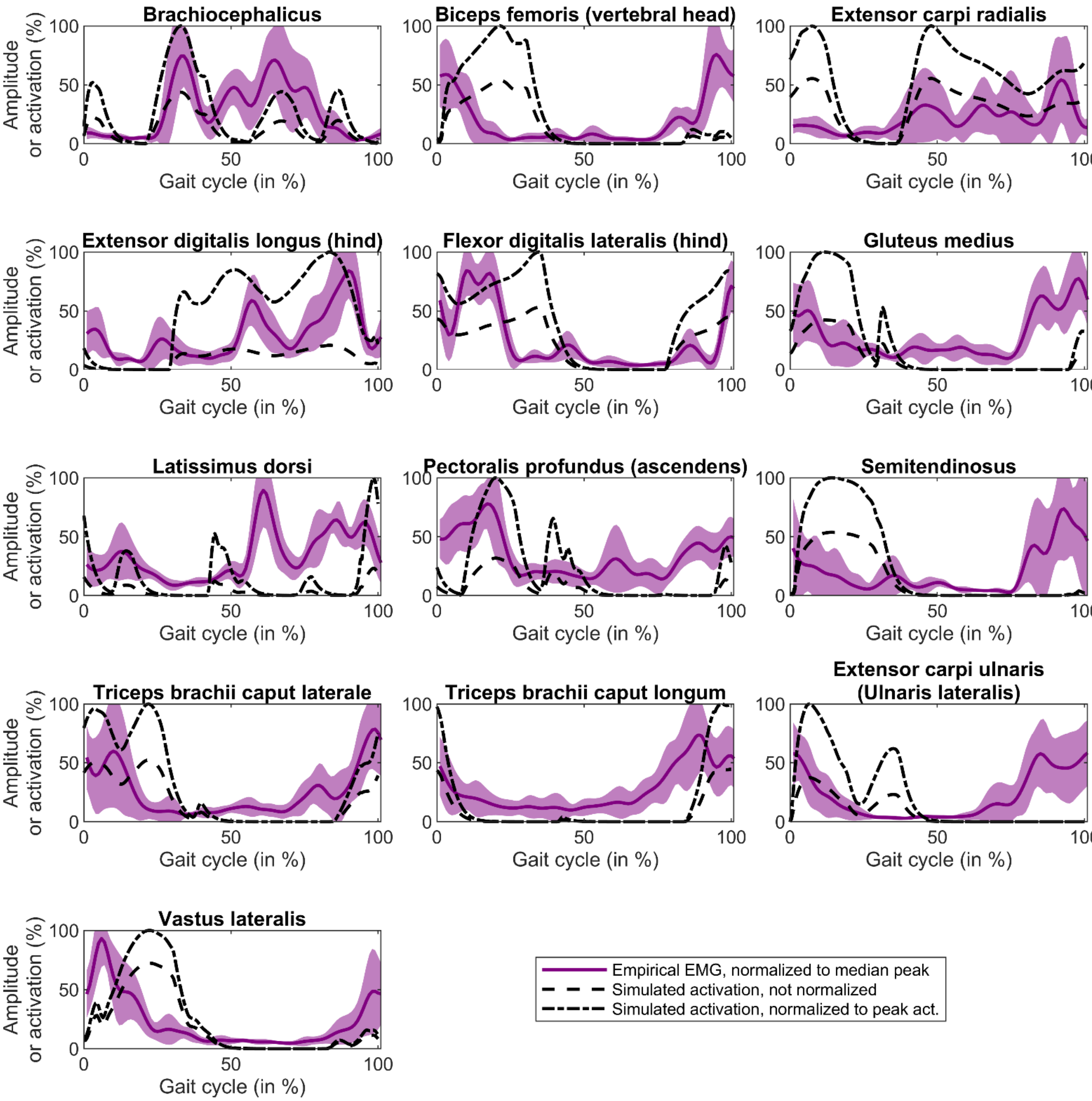

Supplementary Figure S4. Empirical EMG signals from trotting horses (speed range 3.47 – 3.75 m s^{-1}) compared to muscle activations during the kinematic tracking simulations (3 m s^{-1}). All data are synchronized to initial contact of the limb the muscle is attached to. The EMG signals were normalized to the median peak during the stride, and to facilitate comparisons of simulated activations, we plot the simulated activations both with and without normalization to the peak value during the stride (see methods). EMG data from Smit et al. (2024)

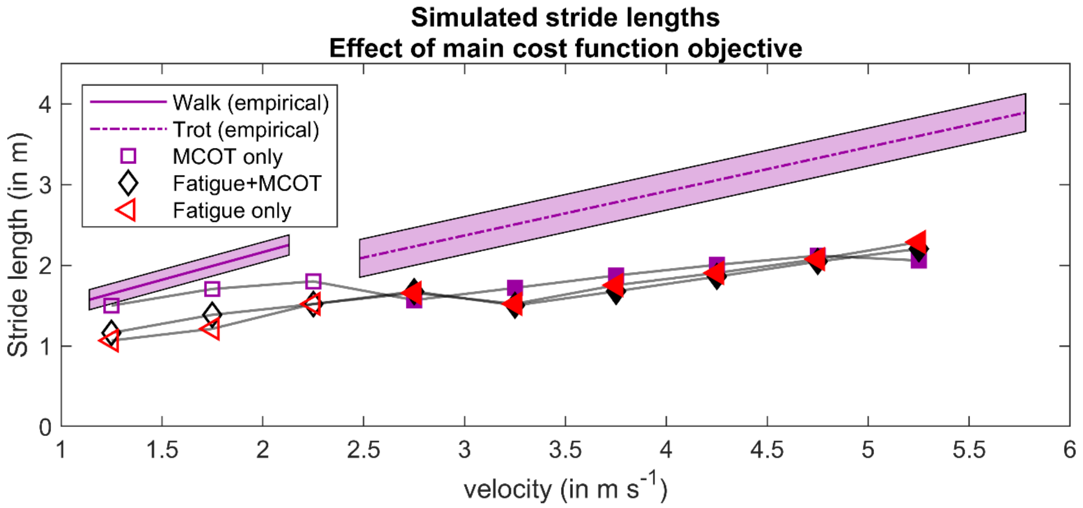

Supplementary Fig. S5. The effect of different cost function weightings on the stride lengths acquired in our feedforward controlled simulations (see Supplementary Texts). Adding Fatigue (parametrized as excitations cubed) tended to decrease stride lengths, whether MCOT was included in the cost function or not. Solid symbols signify running gaits in the same sequence. The “MCOT only” sequence is the same walk to pace sequence that is plotted in Fig. 6 A, main manuscript. Empirical data from Weishaupt et al. (2010).

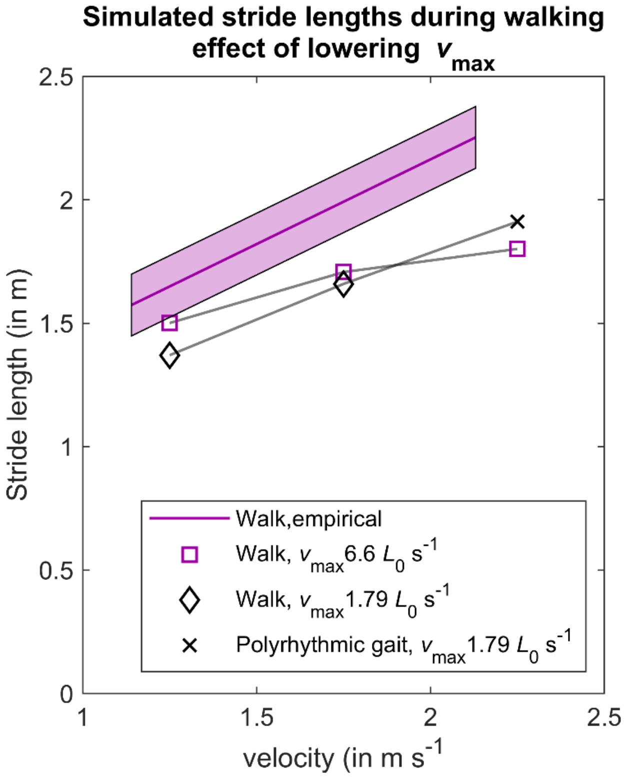

Supplementary Fig. S6. The effect of reducing v_{max} on stride lengths during walking speeds. A v_{max} of 1.79 L_0 s^{-1}, representing slow Type-I fibers, resulted in slightly lower stride lengths at walking speeds (using the same initial guess). It also resulted in the model adopting a polyrhythmic gait when raising the target speed to 2.25 m s^{-1}, this was a local optimum where the hindlimbs contacted twice for each forelimb ground contact. Empirical data from Weishaupt et al. (2010).

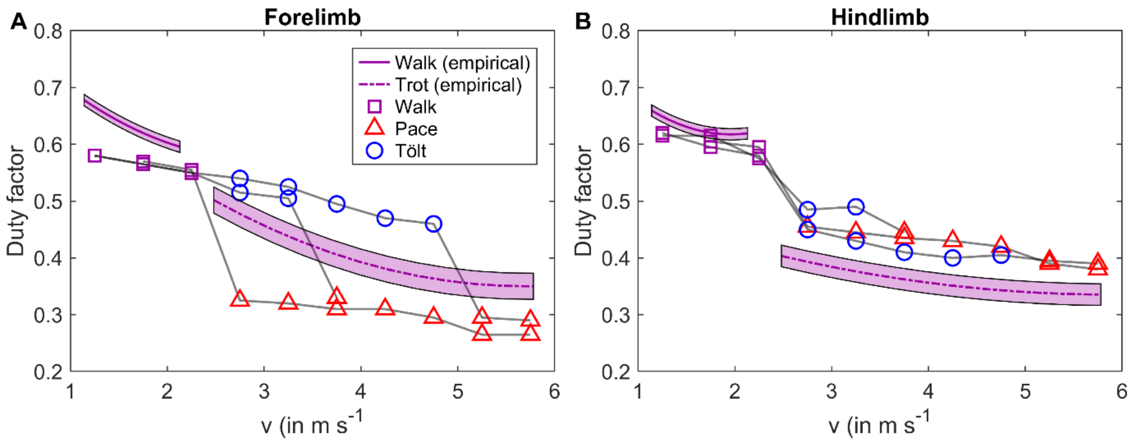

Supplementary Fig. S7. Duty factors of the gait sequences presented in Fig. 6. (A) Forelimb duty factors. The forelimbs used lower duty factors than empirical data, except during locally optimal (reverse) tölting sequences (Fig. 5), which can be interpreted as maintaining forelimb walking dynamics at running speeds. (B) Hindlimb duty factors tended to be lower than empirical duty factors during walking, and higher during running gaits. Empirical data from Weishaupt et al. (2010).

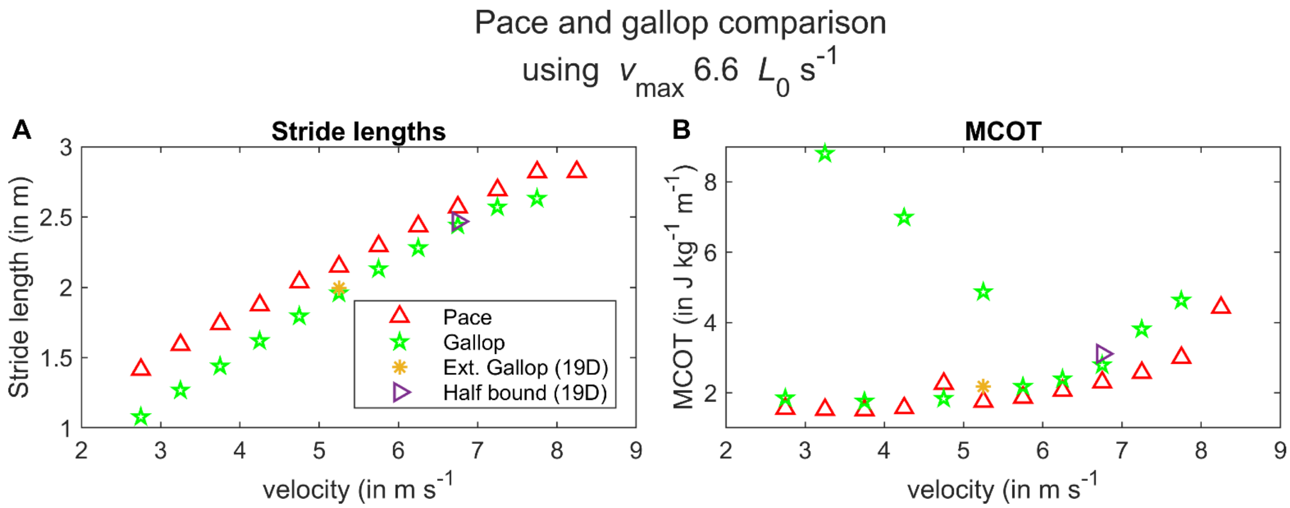

Supplementary Fig. S8. (A) Stride lengths and (B) MCOT over a wide range of simulated running speeds during pacing (Fig. 3) and collected gallop (Fig. 4). All the simulations in this figure used a v_{max} of 6.6 L_0 s^{-1} and minimized MCOT as the main objective. Pacing gaits had higher stride lengths than galloping gaits, and were more economical at all speeds except 4.75 m s^{-1}. Top pacing gait was higher than galloping. Over the range of 3.25 – 5.25 m s^{-1}, we found many locally optimal gaits. We plot two extra simulations where the MTP joint was unlocked (resulting in 19 degrees of freedom). This did not result in substantially longer stride lengths, and MCOT was higher than during pacing at the same speed.

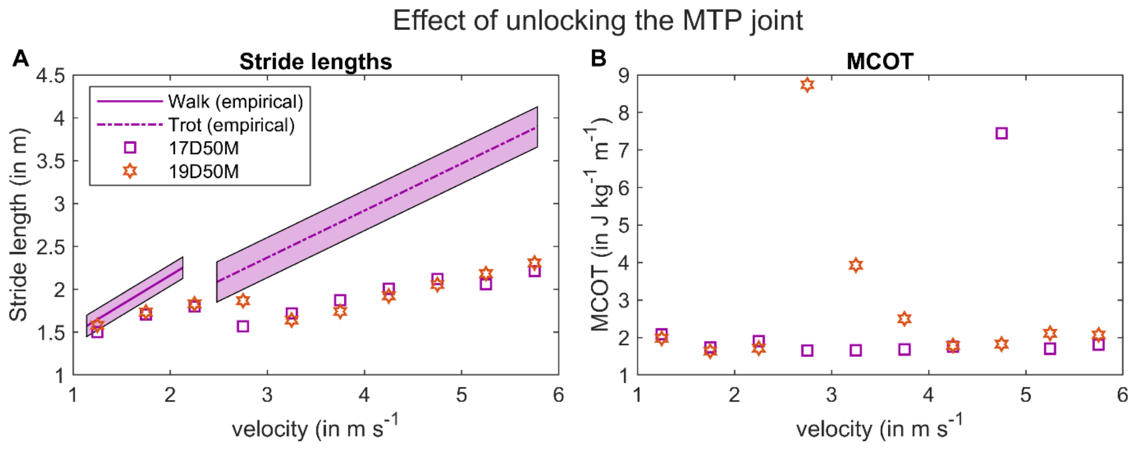

Supplementary Fig. S9. Effect of unlocking the MTP joint on stride lengths (A) Stride lengths and (B) MCOT over a wide range of simulated running speeds. All the simulations in this figure used a v_{max} of 6.6 L_0 s^{-1} and minimized MCOT as the main objective. The 17D50M sequence (purple squares) are the same as the walk-to-pace sequence in Fig. 6. The 19D50M model had an unlocked MTP joint. Empirical data from Weishaupt et al. (2010).

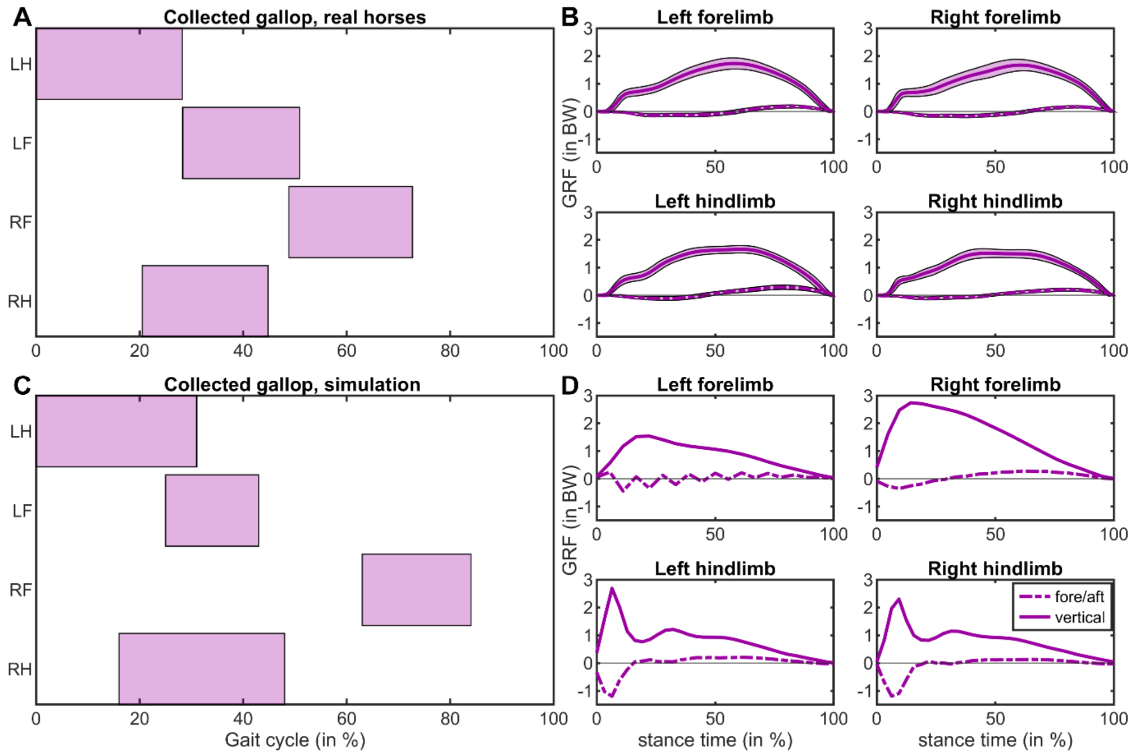

Supplementary Fig. S10. (A) Footfalls (at 9 m s^{-1}) and (B) GRFs (speed range 9.1 – 13.7 m s^{-1}) empirically determined in galloping horses compared to (C) footfalls and (D) GRFs in our model (9.75 m s^{-1}). This simulation was acquired by raising v_{max} from 6.6 to 16 L_0 s^{-1}, using explicit multibody dynamics, and with MCOT as the main objective in the cost function (see Sensitivity Analysis 2 in the supplementary texts). The simulations using explicit multibody dynamics (without the "smooth motions" term in the cost function) enabled higher top speeds, but these gaits were more impulsive at initial contact (hindlimb GRFs), and were more prone to oscillations in the ground contacts (left forelimb GRF). Empirical footfalls were digitized from Witte et al. (2006), and empirical GRFs were digitized from Davies et al. (2019)
