# Supplementary material for: Muscle-Driven Predictive Physics Simulations of Quadrupedal Locomotion in the Horse
Source: Integr Comp Biol. 2024 Jul 13;64(3):694–714. doi: 10.1093/icb/icae095 (PMC11428545; doi:10.1093/icb/icae095)
Supplement: icae095_Supplemental_Files [file icae095_supplemental_files.zip › Supplementary Texts.pdf]

Muscle-driven predictive physics simulations of quadrupedal locomotion in the horse

Authors:
Pasha A. van Bijlert[1,2,*], Thomas Geijtenbeek[3], Ineke H. Smit[4], Anne S. Schulp[1,2], Karl T. Bates[5]

* Correspondence: a.m.vanbijlert@uu.nl; pasha.vanbijlert@naturalis.nl

Supplementary texts

This document contains the following supplementary texts:

Inertial parameters (page 2)
Muscle model components (page 3)
Tuning of musculotendon properties (page 3)
Model joint angles (page 5)
Ground contact parameters (page 5)
Direct collocation, optimizer settings and cost function (page 6)
Tracking simulation (page 7)
Convergence times of the feedforward and feedback controlled simulations (page 7)
Maximal contraction speeds of horse muscle fibers (page 7)
Sensitivity analysis 1: Increasing v_{max} and modifying the cost function for galloping gaits (page 8)
Sensitivity analysis 2: Lowering v_{max} at walking speeds (page 9)

References (page 9)

Inertial parameters

3D inertial properties for our model were estimated by scaling the Dutch Warmblood horse dataset from Buchner et al. (1997) to our horse skeleton. We placed markers on our skeleton (Supplementary Fig. S1) according to figure 1 in Buchner et al. (1997). Perfect correspondence was impossible because not all the markers were defined with respect to bony landmarks, leading to a small amount of uncertainty (see below). We used the marker distances to compute an average scaling factor L = 1.011. This is a linear scale factor between the measurements from Buchner et al. and our model (thus, our horse is slightly larger than their average). L was computed using all segments except the pasterns, hooves (fore/hind) and combined shoulder segments (these were excluded because their lengths were strongly pose-dependent and/or affected by the presence of a hoof). Segment masses M were scaled assuming $M \propto L^3$, moments of inertia I were scaled assuming $I \propto L^5$.

We reconstructed segment centers of mass in Buchner et al.'s body-fixed frames, and then transformed these to an inertial reference frame (see Fig. 1 in the main manuscript for axes directions). Axial segments were assumed to lie perfectly in the midline. We shifted some of the segment centers of mass in lateral direction to ensure they were inside the segments: scapula (0.04 m), humerus (0.04 m), metacarpus (0.015 m), finger (0.03 m), and toe (0.025 m). Moment of inertia tensors were transformed to the global reference frame (in the reference posture, see Fig. 1). Product moments I_{xz} and I_{yz} were set to zero for the axial bodies, because we assume perfect symmetry, and were multiplied by -1 for the right side limb segments. Inertia for the tail body was estimated by sculpting a 3D volume along the tail skeleton, since Buchner et al. (1997) did not it report it. Final inertial parameters are reported in Supplementary Data File S1, sheet 1.

There was some ambiguity to the marker definition in Buchner et al., because they were not always placed on bony landmarks. Marker placement was also described as "usually the lateral projection of the rotation centres of the joints on the skin", but for instance marker 13 (corresponding to greater trochanter of the femur) appears to lie caudal to the hip joint center in their figure.

The segment-based reference frames described in Büchner et al. (1997) are also not wholly unambiguous: local x-axes originate in the proximal markers of each segment, and point towards the distal markers (thus, the x-axis is tangential to the skin/surface of the segment, not through the segment center); local z-axes appear to be defined as orthogonal to the global sagittal plane ("usually the proximal or distal **medial projection** of the joint rotation centre", Buchner et al. (1997), emphasis ours). These axes cannot be mutually orthogonal, because their definition of the x-axes do not lie perfectly on the global XY-plane. If the authors did not intend the local z-axes to be orthogonal to the sagittal plane, then fully reconstructing these coordinate systems is still impossible because the medial markers are not explicitly defined or figured. Our scale factor L = 1.011 was computed including the z-coordinates (in our global reference frame) of the markers. All other computations were performed using the sagittal projections, because without explicit definition of the medial markers, we considered it parsimonious to treat the z-axes as orthogonal to the sagittal plane. Buchner et al. (1997) point out that ignoring off-sagittal conformations will lead to errors in the inertia tensors "within about 5% difference" for most segments, and that this is thus a reasonable simplification.

As an extra exploration on the magnitude of this error, we recomputed L using only the sagittal projections of the markers (L = 1.006). This would lead to a ~1.53% errors in mass, and ~2.59% errors in I. These are well within the standard deviations reported by Buchner et al., and it is likely that treating the segments of an animal as rigid bodies incurs larger (immeasurable) errors than those

caused by the ambiguous body-fixed frames. Lastly, Buchner et al. reported fluid (blood) loss during the dissections as 2.4% body mass, and we did not account for this because it is impossible to determine how this should be distributed between the arm and torso segments.

Muscle model components

The muscles in our model are standard, three-element Hill-type muscles (Hill 1938; Zajac 1989). Muscle fibers are represented by a contractile element (CE) and a parallel elastic element (PEE). The CE has a force-length relationship, maximal isometric force can be generated when the CE is at its optimal fiber length (L_0, in meters). The CE also has a hyperbolic force-velocity relationship, the fastest (unloaded) contraction speed is parametrized by v_{max} (in L_0 s^{-1}). The PEE behaves like a non-linear spring that generates a force when $L_{CE} > L_0$. The CE and PEE are placed in series with a serial elastic element (SEE). This is another non-linear spring with resting length L_T (in meters), which represents the combined elasticity of external and internal tendons.

We used different muscle models for the two types of simulations. We used the model described by De Groote et al. (2016) in the direct collocation simulations because it was specifically optimized (tanh-smoothed) for increased performance when using direct collocation and gradient-based optimizers (De Groote et al. 2016; Dembia et al. 2020). In the feedback-controlled simulations, we used the muscle model described by (Millard et al. 2013). The Millard muscle model is widely used in forward dynamics simulation, because the damping parameter results in a clearly defined inverse force-velocity relationship at zero activation levels. Both muscle models are similar 3-element Hill-type muscle models (Hill 1938; Zajac 1989), and we expect only small differences in their mechanical behavior, given that we kept the important biological inputs (L_0, F_{max}, L_T) constant.

Tuning of musculotendon properties

In terrestrial animals, fiber lengths (and thus implicitly, tendon lengths) are somewhat tuned to specific joint ranges used by animals during locomotion (Burkholder and Lieber 2001). Simulation models can be quite sensitive to these lengths: fascicle lengths measured using dissections do not (always) account for sarcomerial overlap (Burkholder and Lieber 2001) or fiber length distributions (Charles et al. 2022), internal tendons lengths are often not reported (e.g., (Brown et al. 2003; Payne, Hutchinson, et al. 2005)), the onset of *rigor mortis* further complicates length measurements (Burkholder and Lieber 2001; Galton and Shepherd 2012), and simple path actuators can cause errors in fiber excursions (Blemker and Delp 2006). In humans, these uncertainties can be circumvented using joint dynamometry to establish at what joint ranges the muscles function optimally, which is currently infeasible for most animals. Thus, while muscle architectural datasets can provide valuable anatomical insights (Brown et al. 2003; Payne, Hutchinson, et al. 2005; Payne, Veenman, et al. 2005; Watson and Wilson 2007), directly using the reported fiber lengths can cause severe tuning issues in simulation models (van den Bogert et al. 1998), even if the measurements are taken from the same individual that is being modelled. This problem can be somewhat ameliorated by either adjusting only the tendon lengths (Rankin et al. 2016), or both the fiber and tendon lengths (Sellers et al. 2013), to ensure peak muscle forces occur over reasonable joint ranges.

The method we applied here is heavily inspired by Sellers et al. (2013). Essentially, we prescribe a set of joint ranges, and define L_0 as a fraction of the total length change in the muscles when the model moves through these joint ranges. Sellers et al. (2013) proposed using 0.75 – 1.25 L_0 for all the muscles, for a given (locomotor) joint range. This corresponds to an extension length to L_0 ratio of 0.5 (L_0 is twice the change in musculotendon length for a given change in joint angle). In a set of pilot simulations, we experimented extensively using both different combinations of joint ranges, and

different ranges for this length ratio. Using the same ratio for all the muscles resulted in an ill-tuned model. If the ratio was set to 1 (i.e., fibers tuned to function between 0.5 – 1.5 L_0), the proximal muscles appeared to limit joint excursions in simulations. If the ratio was set to 0.5 (fibers tuned between 0.75 – 1.25 L_0), distal muscle fibers became exceedingly long, which per equation (1) results in very low F_{max}. This problem can be avoided by either changing the joint tuning ranges, or changing the extension length to L_0 ratio. We opted for the latter, because our model has non-constant moment arms, and keeping moderately wide joint tuning ranges implicitly accounts for this.

Our final model used the following joint tuning ranges (see Fig. 1a for the neutral position of all the joints): -10° to 10° (scapulothoracic, SCT), -15° to 35° (shoulder), -15° to 50° (elbow), -60° to 0° (wrist), -15° to 40° (hip), -40° to 20° (knee), and 0° to 40° (ankle). Hip, SCT, and mono-articular shoulder crossing muscles were tuned using 0.75 – 1.25 L_0. All other (more distal) muscles were tuned using 0.5 – 1.5 L_0. L_T for most muscles was tuned by determining the musculotendon lengths at the midpoint of the joint tuning ranges, and then subtracting L_0. Thus, we do not account for tendon strain.

We manually retuned three muscles, because we suspected they would otherwise negatively impact the performance of our model. L_0 of the knee flexor ankle extensor muscle (KFAE, modelled after M. gastocnemius caput laterale) was halved, to provide a better match to reported fiber lengths. Tendon lengths of the two bi-articular elbow and wrist crossing muscles (elbow extensor wrist flexor, EEWF, modelled after M. extensor carpi ulnaris; elbow flexor wrist extensor, EFWE, modelled after M. extensor carpi radialis) were tuned at -15° (elbow) and -60° (wrist), to encourage passive coupling between the elbow and wrist joints. This was motivated by the observation that when the carpus was manually extended in a live horse by its handler, full carpal extension was resisted when the elbow was in moderate flexion. There exists ex-vivo experimental evidence that the elbow and MCP joints are coupled (Riemersma et al. 1996), implicitly suggesting that the elbow and wrist are also coupled since the MCP flexors and extensors all cross the wrist. Passive elastic forces are also thought to play a large role in the equine hindlimb (Van Den Bogert et al. 1988), but we did not manually retune the hindlimb further, because there were 5 ankle crossing muscles, and we expected retuning effects to be more unpredictable here than when retuning two wrist-crossing muscles. Final contractile parameters are reported in Supplementary Data File S1, sheet 5, where we also report average & reported ranges of fiber lengths from the literature where available.

L_0 of most muscles fall within the reported ranges, when accounting for the fact that our functional groups are meant to combine the effects of different muscles, and L_0 thus should represent the combined effect of multiple muscles. For example: the hip extensor knee flexor / HEKF, modelled after M. gracilis, has an L_0 that more closely matches the pelvic head of Semimembranosus; hip flexor knee extensor / HFKE, modelled after M. tensor fasciae latae, has a much larger L_0, but falls within the range of sartorius; the hip extensor / HE, modelled after M. gluteus medius, has an L_0 that falls within the range of all the gluteal muscles (Payne, Hutchinson, et al. 2005). Elbow extensor wrist flexor / EEWF, modelled after M. ulnaris lateralis, has an L_0 that falls between M. ulnaris lateralis and M. flexor carpi radialis (Brown et al. 2003). The shoulder extensor / SE, modelled after M. supraspinatus, and the shoulder flexor elbow extensor / SFEE, modelled after M. triceps brachii caput longum, fall outside the empirical ranges in Supplementary Data File S1, sheet 5. However, Watson and Wilson (2007) did not report the range in fascicle lengths within individual muscles, so we reported the range in individual averages, which is a narrower range.

There are some exceptions where a muscle in our model has a more limited role than in real horses. For example, the scapular retractor / ScR muscle, modelled after M. serratus ventralis cervicis, also serves as a cervical extensor in real horses, but not in our model.

Digital flexion was not one of the explicit muscle functions we modelled, but our choice of functional groups resulted in an actuated MTP joint. We present several simulations where we unlocked the MTP joint in our model, resulting in 19 degrees of freedom (19D50M). In these simulations, we reduced the length of the "Ankle extensor, toe flexor" muscle, modelled after M. flexor digitalis lateralis, by 0.04 m, to account for the fact that the above joint tuning ranges did not incorporate MTP flexion or extension. Results from these simulations are presented in Supplementary Fig. S8 and S9.

Model joint angles

Our model was constructed in a neutral, standing posture (Fig. 1). In our base-model, which we have used for all the simulations in this study, the joint angles were defined to be zero in this posture. To facilitate future comparisons of kinematics when using our model, we also provide two alternative versions of our model, where the entire limb is vertically stacked when the joint angles are zero (a posture which a real horse cannot achieve, due to bony and soft tissue constraints). These model versions are provided on our project page. We also provide the joint angle deviations required to achieve a vertically stacked posture in our base-model here: -26.9° (SCT), 57.0° (shoulder), -24.7° (elbow),-7.51° (wrist), -35.0° (MCP), -7.45° (hip), 33.5° (knee), -21.9° (ankle), -37.1° (MTP).

Ground contact parameters

Our model has five contact spheres per limb (radius 0.015 m), arranged in a horseshoe shape (see Supplementary Fig. S1) by aligning radiographs from ("Imaging Anatomy" 2023). Both our simulation modalities used a contact model that incorporates Herz stiffness, a Hunt-Crossley dissipation model, and a Stribeck friction curve (Sherman et al. 2011; Serrancoli et al. 2019). The contact parameters were found through experimentation and formed a compromise between too stiff (which gives numerical issues) and too compliant (which allows too much contact deformation). This approach has been suggested before in the context of equine gait simulations (van den Bogert et al. 1989).

For our feedforward-controlled (direct collocation) simulations, we used the "SmoothSphereHalfSpaceForce" contact model in OpenSim. This is model is smoothed using tanh functions to improve performance in gradient optimizations (Serrancoli et al. 2019). We set plane strain modulus to 11.25 MPa, static and dynamic friction coefficients to 0.4, viscous friction to 0.1. We initially set dissipation to 1 s m^{-1}. The smoothing in the contact model is controlled through two parameters: we set "Hertz_smoothing" to 600 and "hunt_crossley_smoothing" to 50. The Hertz smoothing essentially results in (small) forces being generated before contact penetration (Serrancoli et al. 2019). Conversely, the Hunt-Crossley smoothing can cause negative contact forces at certain penetration speeds, which can lead to situations where there is ground contact but no contact force. In the direct collocation simulations, the smoothing in the contact model resulted in issues (no contact force despite contact penetration) at target speeds beyond walking speed (1.25 m s^{-1}). At higher target speeds, we resolved this by lowering contact dissipation to 0.1 s m^{-1} (which is close to the optimized value found in human sprinting simulations (Haralabidis et al. 2021)).

For the feedback-controlled simulations, we used the "contact_force_hunt_crossley_sb" contact model in Hyfydy. Although the implementation is similar, this contact model does not include any smoothing, and the parameter that decides the contact-stiffness is recomputed to be contact-sphere radius independent (see the Hyfydy documentation). In Hyfydy, we set the stiffness-parameter to 15 kN $m^{-3/2}$ (effectively identical to the direct collocation simulations). Friction parameters were kept the same.

Direct collocation, optimizer settings and cost function

It is currently not fully-known how animals select their gaits, although it is often assumed that muscle activity (and by extension, energy cost) is among the main factors (Ackermann and van den Bogert 2010; Falisse et al. 2019; McDonald et al. 2022). Similar to Falisse et al. (2019), we have used a multi-objective cost function. We have described and discussed this cost-function in detail in an unrelated publication on emu gait simulations (van Bijlert et al. 2024) (in-review), and reused it here.

$$C = \int_0^T (\underbrace{w_1 \frac{\sum_{m=1}^{N_\mathrm{M}} |U_m|^3}{N_\mathrm{M}\, d}}_{\text{Fatigue}} + \underbrace{w_2 \frac{\sum_{m=1}^{N_\mathrm{M}} \dot{E}_m}{M_\mathrm{Tot}\, d}}_{MCOT} + \underbrace{w_3 \frac{\sum_{m=1}^{N_\mathrm{M}} \dot{F}_{\mathrm{T},m}^2}{N_\mathrm{M}\, v_{\mathrm{p},x}}}_{\text{Smooth contractions}} + \underbrace{w_4 \frac{\sum_{i=1}^{N_\mathrm{I}} \ddot{q}_i^2}{N_\mathrm{I}\, v_{\mathrm{p},x}}}_{\text{Smooth motions}})\, \mathrm{d}t$$

$w_1 – w_4$ = weights that scale relative contributions of the terms
T = final time point (in s) of the simulation
N_M = number of muscles in the model (i.e., functional groups)
N_I = number of coordinates (degrees of freedom)
U_m = control input, or excitation (bounded between 0.0001 and 1), of muscle m
d = distance traversed (in m) in x-direction by the pelvis, between $t = 0$ and $t = T$
$v_{\mathrm{p},x}$ = average velocity (in m s^{-1}) in x-direction of the pelvis
M_Tot = total body mass (in kg)
$\dot{E}_m$ = total metabolic rate (in W) of muscle m
$\dot{F}_{\mathrm{T},m}$ = time-derivative of the normalized tendon force of muscle m
$\ddot{q}_i$ = second time-derivative of coordinate

The two main costs relate to muscle fatigue (Ackermann and van den Bogert 2010), and metabolic cost of transport (MCOT, in J kg^{-1} m^{-1}), using the phenomenological model of Bhargava et al. (2004)). Their relative weights w_1 and w_2 were scaled so that relative contributions were approximately equal at 1.25 m s^{-1}, because this is close to the optimal walking speed of ponies and horses (Hoyt and Taylor 1981; Minetti et al. 1999). The smoothness criteria were optional, secondary costs, scaled to a much lower weight. These are mainly there to improve convergence (Falisse et al. 2019), although they bias the optimizations towards smoother gaits – which can be interpreted as phenomenologically modelling force / pressure sensing in the muscles and limbs.

Moco allows explicit and implicit formulations for the differential equations describing the multibody (MBD) and tendon dynamics (Dembia et al. 2020). In Moco, the smoothness criteria can only be added to the cost function when using implicit MBD and tendon dynamics, which adds extra states to the optimization problem. When searching for walking gaits using a quasi-random initial guess, we had the most success with explicit MBD and rigid tendons (no tendon dynamics). Our final solutions always used elastic tendons (with implicit dynamics, and thus the “smooth contractions” criterion). Explicit multibody dynamics (without the “smooth movements” criterion) enabled slightly higher top speeds in our model, but this resulted in impulsive GRFs (see sensitivity analysis 1). Our code examples show how we implemented the cost function in Matlab.

We set convergence tolerance in Moco to 10^{-3}, and constraint tolerance to 10^{-4}. The trajectories were discretized into 101 timepoints.

Tracking simulation

We performed one simulation where squared deviations from empirical joint angles were added as a tracking term to the cost function. This term can be written as follows:

$$C_{\mathrm{T}} = \int_0^T w_5 \sum_{j=1}^{N_{\mathrm{J}}} \left(\Phi_j - \varphi_j\right)^2 \, \mathrm{d}t$$

C_{T} = Tracking cost
w_5 = weighting term
N_{J} = number of joints that are being tracked
Φ_j = empirical joint angle *j*
φ_j = model joint angle *j*

We used the joint angles presented in (Back et al. 1995a, 1995b) of a Dutch warmblood horse trotting at 3.0 m s^{-1}. Back et al. corrected these joint angles for skin-displacements following (van Weeren et al. 1992), making this a suitable comparison for our model. The joint angles as presented in those publications are deviations with respect to the joint angles they measured when the horse was standing (Back et al. 1995a, 1995b), and they did not provide absolute segment angles of these standing postures. Thus, it was only possible to track angle deviations, furthermore under the assumption that our reconstructed standing posture (Fig. 1) was close to the empirical measurements.

Similar to (Fox 2024), we used the empirical joint angles as the initial guess for the simulation. This simulation was performed using both MCOT and Fatigue, to encourage force-sharing across the muscles (Ackermann and van den Bogert 2010). We have provided the full code for this tracking simulation on our project page.

Convergence times of the feedforward and feedback controlled simulations

All of our simulations were performed on a computer with an AMD Ryzen 9 5900x 12-core 3.7 GHz processor. We have timed the example scripts on our project page. In OpenSim Moco, the 11D24M model converged in 52 minutes (923 iterations) using a quasi-random initial guess, and the 17D50M model converged in 266 minutes (598 iterations) using an 13D24M trajectory as an initial guess. In Hyfydy, reinitializing the 17D50M model from a previously converged optimum, a single optimization converged in 20 minutes (57480 evaluations, using a 20 second simulation and a population size of 20). We caution the reader that these convergence times are not directly comparable – finding an adequate gait solution using the feedback controller requires extensive searching with multiple parallel optimizations.

Maximal contraction speeds of horse muscle fibers

v_{max} scales negatively with body size (Rome et al. 1990; Medler 2002). This is thought to represent a tuning mechanism, where the energetically optimal contraction speed of muscles is somewhat matched to the (natural) frequencies of relevant locomotor body segments (Hill 1950; Medler 2002). Due to the force-length-velocity relationship of muscle tissue, maximal (energetic) efficiency occurs at a lower contraction speed (0.2 v_{max}) than maximal power output (0.3 v_{max}) (Hill 1938, 1950). Limbs of larger animals will have lower natural frequencies, and a lower v_{max} ensures that muscles can still contract near their energetic optimum. It is thus necessary to choose an appropriate v_{max} for our horse model.

v_{max} of equine muscle fibers have been reported in three studies (Rome et al. 1990; Marx et al. 2006; Butcher et al. 2010). Measurements were performed at 15 °C or 30 °C, but Dutch Warmblood horses have a body temperature near 37.5 °C. Contraction speed approximately doubles with every 10 degree increase (Q10 = 2, (Bennett 1984)). We compensated for these temperature differences by scaling reported v_{max} by 5 (=2*2*1.25) and 1.75 for measurements performed at 15 °C and 30 °C, respectively. Original and scaled data are presented in Supplementary Data File S1, sheet 7. Reported values for equine v_{max} of Type I and IIa fibers thus differ by a factor of ~2 (Rome et al. 1990; Marx et al. 2006). It is unclear what explains this difference, although it could be due to the different muscles studied (soleus in Rome et al. (1990) versus gluteus medius in Marx et al. (2006)). The equine soleus is typically characterized by predominantly slow Type 1 fibers (86% (Kawai et al. 2009) to 100% (Butcher et al. 2010)), whereas gluteus medius only contains 5-14% Type I (the rest is approximately evenly distributed between fast Type IIa and IIx (Kawai et al. 2009)). Given the closer match between Rome et al. (1990) and Butcher et al. (2010), it is possible that some of the measurements in Marx et al. (2006) are faster because their measurements were in fact of mixed Type I, IIa and IIx fibers, but reported as Type I and Type IIa. This interpretation is plausible based on Fig. 2 of their manuscript, where horse muscles were observed to have a large fraction of Type IIx fibers (Marx et al. 2006).

By combining average fiber compositions of horse muscles in Kawai et al. (2009) with temperature-corrected v_{max} of individual fiber types (Supplementary Data File S1, sheet 7) from (Rome et al. 1990), it is possible to compute the v_{max} of an "average" mixed-fiber type muscle: 6.6 L_0 s^{-1}. We used this for most of our simulations. This is somewhat higher than the mixed-fiber type muscles reported by Butcher et al. (2010) (Supplementary Data File S1, sheet 7), potentially because Butcher et al. found no traces of Type IIx fibers. The next two sections report sensitivity analyses where we evaluate the effect of both lowering and raising v_{max}, with physiological justifications.

Sensitivity analysis 1: Increasing v_{max} and modifying the cost function for galloping gaits

Most muscles in the horse are pennate (Payne, Hutchinson, et al. 2005; Payne, Veenman, et al. 2005), and in Hill-type muscle models, changes in pennation angle during contraction would increase the shortening speed of the whole muscle with respect to the fiber (Zajac 1989), even though the fiber's contraction speed along the muscle line of action is lower. Because we did not simulate the effect of muscle pennation in this study, it is possible that we underestimated the "effective" v_{max} for the muscle functional groups. To account for this, we investigated the sensitivity of top galloping speed to this parameter, by trying two higher values than 6.6 L_0 s^{-1}. We performed simulations using 13.3 L_0 s^{-1} and 16 L_0 s^{-1} for all the muscles.

16 L_0 s^{-1} is the highest reported equine v_{max}, temperature corrected as described above (Supplementary Data File S1, sheet 7), and this simulation thus assumes all muscles in our model are parallel fibered muscles with fiber type IIx. 13.3 L_0 s^{-1} is an intermediate value we acquired by multiplying 16 with the cosine of 33.5 degrees (the average of all the pennation angles of the horse hindlimb (Payne, Hutchinson, et al. 2005)). This is the effective contraction speed of a Type IIx fiber with constant pennation angle of 33.5 degrees. These should both be interpreted as wide upper bounds on v_{max}, given that they are based on the assumption that all muscles are Type IIx. Despite this being a very steep increase in v_{max}, top galloping speed did not strongly increase (see next paragraph).

Using v_{max} = 6.6 L_0 s^{-1}, top speed was 7.75 m s^{-1}. Using v_{max} = 13.3 L_0 s^{-1}, top speed was 8.25 m s^{-1}. Using v_{max} = 16 L_0 s^{-1}, top speed was 8.75 m s^{-1} (this result was plotted in the main manuscript). We did not try higher values for v_{max}, and all of these results were found using MCOT as the main cost, while also including both the "smooth motions" and "smooth contractions" criteria (i.e., using both

implicit multibody and tendon dynamics, see "Direct collocation, optimizer settings and cost function"). We were able to achieve slightly higher top speeds when we omitted the "smooth contractions" criterion by using explicit multibody dynamics. For a v_{max} of both 13.3 and 16 L_0 s^{-1}, top speeds increased to 9.75 m s^{-1}. However, these simulations tended to have more impulsive, non-smooth GRFs, see Supplementary Fig. S10.

Sensitivity analysis 2: Lowering v_{max} at walking speeds

Due to the size principle of muscle recruitment (Henneman and Olson 1965), it is reasonable to assume that the fastest (type IIa and IIx) muscle fibers are only recruited during (near-)maximal efforts. During slow walking gaits, where it is likely that mostly slow Type I fibers are preferentially recruited, stride frequencies may thus be determined by the v_{max} of the slow muscle fibers. For target speeds (1.25 – 1.75 m s^{-1}), we reran the simulations with a v_{max} of 1.79 L_0 s^{-1} (Supplementary Fig. S6). This value was based on the average between Type I fibers measurements reported in Rome et al. (1990) and Butcher et al. (2010). These simulations had lower stride lengths than when using a v_{max} of 6.6 L_0 s^{-1}, and transitioned to a polyrhythmic gait at target speeds of 2.25 m s^{-1} and higher. This was a gait where both hindlimbs had two contacts per forelimb contact. We rejected these polyrhythmic gaits as local optima.

References

Ackermann M, van den Bogert AJ. 2010. Optimality principles for model-based prediction of human gait. J Biomech 43:1055–60.

Back W, Schamhardt HC, Savelberg HHCM, Van Den Bogert AJ, Bruin G, Hartman W, Barneveld A. 1995a. How the horse moves: 1. Significance of graphical representations of equine forelimb kinematics. Equine Vet J 27:31–38.

Back W, Schamhardt HC, Savelberg HHCM, Van Den Bogert AJ, Bruin G, Hartman W, Barneveld A. 1995b. How the horse moves: 2. Significance of graphical representations of equine hind limb kinematics. Equine Vet J 27:39–45.

Bennett AF. 1984. Thermal dependence of muscle function. Am J Physiol-Regul Integr Comp Physiol 247:R217–29.

Bhargava LJ, Pandy MG, Anderson FC. 2004. A phenomenological model for estimating metabolic energy consumption in muscle contraction. J Biomech 37:81–88.

Blemker SS, Delp SL. 2006. Rectus femoris and vastus intermedius fiber excursions predicted by three-dimensional muscle models. J Biomech 39:1383–91.

Brown NAT, Kawcak CE, McIlwraith CW, Pandy MG. 2003. Architectural properties of distal forelimb muscles in horses, *Equus caballus*. J Morphol 258:106–14.

Buchner HHF, Savelberg HHCM, Schamhardt HC, Barneveld A. 1997. Inertial properties of Dutch Warmblood horses. J Biomech 30:653–58.

Burkholder TJ, Lieber RL. 2001. Sarcomere length operating range of vertebrate muscles during movement. J Exp Biol 204:1529–36.

Butcher MT, Chase PB, Hermanson JW, Clark AN, Brunet NM, Bertram JEA. 2010. Contractile properties of muscle fibers from the deep and superficial digital flexors of horses. Am J Physiol-Regul Integr Comp Physiol 299:R996–1005.

Charles J, Kissane R, Hoehfurtner T, Bates KT. 2022. From fibre to function: are we accurately representing muscle architecture and performance? Biol Rev 97:1640–76.

De Groote F, Kinney AL, Rao AV, Fregly BJ. 2016. Evaluation of Direct Collocation Optimal Control Problem Formulations for Solving the Muscle Redundancy Problem. Ann Biomed Eng 44:2922–36.

Dembia CL, Bianco NA, Falisse A, Hicks JL, Delp SL. 2020. OpenSim Moco: Musculoskeletal optimal control. PLOS Comput Biol 16:e1008493.

Falisse A, Serrancolí G, Dembia CL, Gillis J, Jonkers I, De Groote F. 2019. Rapid predictive simulations with complex musculoskeletal models suggest that diverse healthy and pathological human gaits can emerge from similar control strategies. J R Soc Interface 16.

Fox AS. 2024. The quest for dynamic consistency: a comparison of OpenSim tools for residual reduction in simulations of human running. R Soc Open Sci 11:231909.

Galton PM, Shepherd JD. 2012. Experimental Analysis of Perching in the European Starling (*Sturnus vulgaris* : Passeriformes; Passeres), and the Automatic Perching Mechanism of Birds. J Exp Zool Part Ecol Genet Physiol 317:205–15.

Haralabidis N, Serrancolí G, Colyer S, Bezodis I, Salo A, Cazzola D. 2021. Three-dimensional data-tracking simulations of sprinting using a direct collocation optimal control approach. PeerJ 9:e10975.

Henneman E, Olson CB. 1965. Relations between structure and function in the design of skeletal muscles. J Neurophysiol 28:581–98.

Hill AV. 1938. The Heat of Shortening and the Dynamic Constants of Muscle. Proc R Soc B Biol Sci 126:136–95.

Hill AV. 1950. THE DIMENSIONS OF ANIMALS AND THEIR MUSCULAR DYNAMICS. Sci Prog 1933-38:209–30.

Hoyt DF, Taylor R. 1981. Gait and the energetics of locomotion in horses. Nature 292:239–40.

Imaging Anatomy. 2023. . (https://vetmed.illinois.edu/imaging_anatomy/index.html).

Kawai M, Minami Y, Sayama Y, Kuwano A, Hiraga A, Miyata H. 2009. Muscle Fiber Population and Biochemical Properties of Whole Body Muscles in Thoroughbred Horses. Anat Rec 292:1663–69.

Marx JO, Olsson MC, Larsson L. 2006. Scaling of skeletal muscle shortening velocity in mammals representing a 100,000-fold difference in body size. Pflüg Arch - Eur J Physiol 452:222–30.

McDonald KA, Cusumano JP, Hieronymi A, Rubenson J. 2022. Humans trade off whole-body energy cost to avoid overburdening muscles while walking. Proc R Soc B Biol Sci 289:20221189.

Medler S. 2002. Comparative trends in shortening velocity and force production in skeletal muscles. Am J Physiol-Regul Integr Comp Physiol 283:R368–78.

Millard M, Uchida T, Seth A, Delp SL. 2013. Flexing computational muscle: modeling and simulation of musculotendon dynamics. J Biomech Eng 135:021005.

Minetti AE, Ardigò LP, Reinach E, Saibene F. 1999. The relationship between mechanical work and energy expenditure of locomotion in horses. J Exp Biol 202:2329–38.

Payne RC, Hutchinson JR, Robilliard JJ, Smith NC, Wilson AM. 2005. Functional specialisation of pelvic limb anatomy in horses (*Equus caballus*). J Anat 206:557–74.

Payne RC, Veenman P, Wilson AM. 2005. The role of the extrinsic thoracic limb muscles in equine locomotion. J Anat 206:193–204.

Rankin JW, Rubenson J, Hutchinson JR. 2016. Inferring muscle functional roles of the ostrich pelvic limb during walking and running using computer optimization. J R Soc Interface 13.

Riemersma DJ, Van Den Bogert AJ, Jansen MO, Schamhardt HC. 1996. Tendon strain in the forelimbs as a function of gait and ground characteristics and *in vitro* limb loading in ponies. Equine Vet J 28:133–38.

Rome LC, Sosnicki AA, Goble DO. 1990. Maximum velocity of shortening of three fibre types from horse soleus muscle: implications for scaling with body size. J Physiol 431:173–85.

Sellers WI, Margetts L, Coria RA, Manning PL. 2013. March of the Titans: The Locomotor Capabilities of Sauropod Dinosaurs. PLoS ONE 8:e78733.

Serrancoli G, Falisse A, Dembia C, Vantilt J, Tanghe K, Lefeber D, Jonkers I, De Schutter J, De Groote F. 2019. Subject-Exoskeleton Contact Model Calibration Leads to Accurate Interaction Force Predictions. IEEE Trans Neural Syst Rehabil Eng 27:1597–1605.

Sherman MA, Seth A, Delp SL. 2011. Simbody: multibody dynamics for biomedical research. Procedia IUTAM 2:241–61.

van Bijlert PA, van Soest AJ “Knoek,” Schulp AS, Bates KT. 2024. Muscle-controlled physics simulations of the emu (a large running bird) resolve grounded running paradox (preprint). bioRxiv 2024.01.17.575928. doi: https://doi.org/10.1101/2024.01.17.575928.

van den Bogert AJ, Gerritsen KGM, Cole GK. 1998. Human muscle modelling from a user’s perspective. J Electromyogr Kinesiol 8:119–24.

Van Den Bogert AJ, Hartman W, Schamhardt HC, Sauren AAHJ. 1988. In vivo relationship between force, EMG and length change in the deep digital flexor muscle of the horse. In: de Groot G, Hollander AP, Huijing PA, editors. Biomechanics XI : XI international symposium of biomechanics Presented at the International Society of Biomechanics Congress. Amsterdam: Free University Press. p. 68–74.

van den Bogert AJ, Schamhardt HC, Crowe A. 1989. Simulation of quadrupedal locomotion using a rigid body model. J Biomech 22:33–41.

van Weeren PR, van den Bogert AJ, Barneveld A. 1992. Correction models for skin displacement in equine kinematics gait analysis. J Equine Vet Sci 12:178–92.

Watson JC, Wilson AM. 2007. Muscle architecture of biceps brachii, triceps brachii and supraspinatus in the horse. J Anat 210:32–40.

Zajac FE. 1989. Muscle and Tendon: Properties, models, scaling and application to biomechanics and motor control. Crit Rev Biomed Eng 17:359–408.
